# Identification and Characterization of Blood and Neutrophil-Associated Microbiomes in Patients with Severe Acute Pancreatitis Using Next-Generation Sequencing

**DOI:** 10.3389/fcimb.2018.00005

**Published:** 2018-01-23

**Authors:** Qiurong Li, Chenyang Wang, Chun Tang, Xiaofan Zhao, Qin He, Jieshou Li

**Affiliations:** Research Institute of General Surgery, Jinling Hospital, Medical School, Nanjing University, Nanjing, China

**Keywords:** blood microbiome, neutrophil-associated microbiome, severe acute pancreatitis, sepsis, next-generation sequencing, proteomics

## Abstract

Infectious complications are a leading cause of death for patients with severe acute pancreatitis (SAP). Yet, our knowledge about details of the blood microbial landscape in SAP patients remains limited. Recently, some studies have reported that the peripheral circulation harbors a diverse bacterial community in healthy and septic subjects. The objective of this study was to examine the presence of the blood bacterial microbiome in SAP patients and its potential role in the development of infectious complications. Here we conducted a prospective observational study on a cohort of 50 SAP patients and 12 healthy subjects to profile the bacterial composition in the blood. The patients were subgrouped into uninfected (*n* = 17), infected (*n* = 16), and septic (*n* = 17) cases. Applying 16S rDNA-based next-generation sequencing technique, we investigated blood and neutrophil-associated microbiomes in SAP patients, and assessed their connections with immunological alterations. Based on the sequencing data, a diverse bacterial microbiota was found in peripheral blood and neutrophils from the healthy and SAP subjects. As compared to healthy controls, the blood and neutrophil-associated microbiomes in the patients were significantly altered, with an expansion in Bacteroidetes and Firmicutes as well as a decrease in Actinobacteria. Variations in the microbiome composition in patients were associated with immunological disorders, including altered lymphocyte subgroups, elevated levels of serum cytokines and altered proteomic profiles of neutrophils. However, no significant compositional difference was observed between the patient subgroups, implying that the microbiota alterations might not be linked to presence/absence of infectious complications in SAP. Together, we present an initial description of the blood and neutrophil-associated bacterial profiles in SAP patients, offering novel evidence for the existence of the blood microbiome. Identification of the blood microbiome provides novel insights into characteristics and diagnostics of bacteremia in the patients. Further study is required to assess the possible implications of the blood microbiome in health and diseases.

## Introduction

Severe acute pancreatitis (SAP) is a terrible disease, associated with a mortality rate in the range of 20–50% (Nathens et al., 2004; Garg et al., 2005; Noor et al., 2011). Among the cases with SAP, up to 80% of deaths are attributed to infectious complications and multiple organ dysfunction syndromes (MODS) (Garg et al., 2005). Despite antibiotic prophylaxis in the management of SAP, the incidence of systemic infections is still surprisingly high (Villatoro et al., 2010). Infectious complications have become a major concern in SAP, especially cases of pancreatic necrosis (Medich et al., 1993). Accumulating evidence has suggested that systemic infections in SAP patients are mainly derived from invasion by gut organisms (Schmid et al., 1999; Noor et al., 2011). Yet, the molecular mechanisms behind the development of systemic infections in SAP are not fully known.

During the last two decades, bacterial translocation has been thought to be a major source causing systemic infection and MODS in SAP patients (MacFie et al., 1999; Cicalese et al., 2001). Enteric bacteria could cross the impaired intestinal barrier to reach peripheral circulation, leading to infected pancreatic necrosis and sepsis (Ammori et al., 1999). Just like in patients who underwent sepsis following severe trauma, burn, major operative intervention and other causes, cultures of blood specimens in SAP patients complicated by sepsis are often negative, even in the presence of infected pancreatic necrosis (Sainio et al., 1995; Ammori et al., 2003). As a result, specific interventions against infections are probably delayed in some cases, causing lethal complications. It is quite possible that enteric bacteria may translocate into systemic circulation, but escape from detection by standard culture methods. Utilization of new techniques, such as polymerase chain reaction or matrix-assisted laser desorption ionization–time of flight, to some extent, has improved the ability to detect bloodstream pathogens (Ecker et al., 2010; Buehler et al., 2016), however, our knowledge on the blood-microbial landscape in septic patients is still limited. Appling a culture-independent method, we observed that bloodstream invasion by multiple gut organisms (commonly 5–8 bacterial species) contributed to the development of bacteremia in SAP patients (Li et al., 2013a). Owing to insensitivity of this approach to low-abundance microbial sequences (Muyzer et al., 1993), the bacterial taxonomic richness in the blood of the patients was most likely underestimated. As a result, a deeper exploration of the blood bacterial composition and diversity with emerging molecular techniques might be needed. Recent studies with 16S rDNA-based high-throughput sequencing showed that a diverse microbiota is present in the blood of septic patients (Grumaz et al., 2016; Gosiewski et al., 2017) and healthy individuals (Païssé et al., 2016). Although its biological and clinical significance remains to be further explored, discovery of a blood microbiome might represent an important step toward a better understanding of the microbial world of the human body and its relationships with health and diseases. Therefore, it is urgently needed to ascertain whether a rich microbiota is harbored in blood of SAP patients using culture-independent next-generation sequencing techniques to better understand the development of bacteremia and infectious complications.

The complex network of immune cells and specialized molecules has evolved to defend against pathogens. Various types of immune cells, including neutrophils, monocytes, and lymphocytes, could integrate microbial signals to govern the inflammatory response, together maintaining the homeostasis of systemic circulation (Akira et al., 2006). Recent studies have revealed that sepsis is probably the sequelae of a cascade of events starting with a local inflammatory response against organisms derived from the gut (Bosmann and Ward, 2013). When pathogens invade, the innate immune system recognizes microbial molecules and kicks off an inflammatory response (Akira et al., 2006). Activation of neutrophils could induce excessive production of pro-inflammatory cytokines and disrupt the balance of the pro- and anti-inflammatory response, leading to an overwhelming imbalance (Delano et al., 2011; Bosmann and Ward, 2013). As the first-line responder against pathogens, the neutrophils play a central role for elimination of bacterial infection. Some pathogens can be engulfed and gain entry to the cells (Appelberg, 2007), shaping the neutrophil-associated microbiomes (NAMs). The functional impairment of neutrophils could disrupt the dynamic balance between internalization and clearance of pathogens (Amulic et al., 2012; Hotchkiss et al., 2013; Matthew et al., 2016), likely altering the membership of the neutrophil-associated microbiome and causing infection. However, the composition of the community in neutrophils and its role in sepsis remains uncharacterized. Elucidation of changes of the NAMs and the potential connection with host immunological disorders would be helpful for better understanding the mechanism of sepsis pathogenesis in SAP patients.

Here we performed 16S rDNA-based sequencing on the blood and neutrophils of SAP patients to profile the microbial landscape in peripheral circulation and estimated its potential connection with the development of bacteremia and infectious complications. In addition, we dissected the immune cell repertoires in blood and the proteomic profiles of neutrophils through a fluorescence activated cell sorting (FACS) approach and quantitative proteomics analysis, and examined the relationships of blood microbiota alterations with immunological disorders in SAP patients.

## Materials and methods

### Ethics statement

All participants provided written informed consent upon enrollment. Studies were approved by the Human Subjects Institutional Committee of Jinling Hospital and were conducted in accordance with all relevant guidelines and regulations.

### Study populations and experimental design

Fifty patients who underwent SAP and admitted to the Department of General Surgery, Jinling Hospital, between March 2014 and March 2016, were enrolled in this study. Acute pancreatitis was diagnosed in accordance with clinical symptoms and at least 3 times the upper limit of normal value in serum amylase or evidence on computed tomographic scan of the abdomen (Bradley, 1993). SAP is defined as the presence or absence of organ failure and/or local complications, such as pancreatic necrosis, abscess or pseudocyst (Bradley, 1993). Based on the presence or absence of infectious symptoms, the patients were distributed into three subgroups: uninfected (*n* = 17), infected (*n* = 16) and septic (*n* = 17) (Table 1). Septic patients were identified according to the presence of suspected or documented infections and an acute increase in the Sequential (Sepsis-related) Organ Failure Assessment (SOFA) score of 2 points or more (Singer et al., 2016). The infected patients were defined as having suspected or documented infections but an absence of emerging organ dysfunction, and uninfected cases showing no infectious signs. The patients' clinical characteristics, such as demographic data, clinical diagnoses, comorbidities, vital signs, hematologic and chemical data, blood gas analyses, blood cultures, Acute Physiology and Chronic Health Examination-II (APACHE-II) score and SOFA scores, were recorded (Supplementary Table 1). Twelve healthy volunteers who had no infectious signs and no elevated level of serum CRP served as control subjects. Subjects <18 or more than 70 years old were excluded in this study. Blood samples from enrolled patients and healthy subjects were collected for high-throughput sequencing, quantitative proteomics analysis of peripheral neutrophils, and measurement of blood immune cell subpopulations.

**Table 1:** Demographics of study population.

		**Uninfected (*N* = 17)**	**Infected (*N* = 16)**	**Septic (*N* = 17)**	**Healthy (*N* = 12)**
Age		43.1 ± 11.5	40.4 ± 12.1	47.3 ± 10.7	29.2 ± 3.8
Male (%)		9 (53)	10 (63)	11 (65)	10 (83)
Female (%)		8 (47)	6 (37)	6 (35)	2 (17)
APACHE II scores		4 ± 2	9 ± 1	15 ± 3	0
SOFA scores		0.06 ± 0.24	0.44 ± 0.11	6.47 ± 3.45	0
Lac (mmol/L)		1.3 ± 0.5	1.2 ± 0.5	2.4 ± 2.7	N/A
C-reactive protein (mg/L)		59.5 ± 37.1	133.4 ± 77.3	196.1 ± 71.2	N/A
Hematologic analysis	White blood cell count (×10^9^/L)	8.7 ± 2.5	12.8 ± 7.5	16.9 ± 14.6	6.7 ± 1.1
	Neutrophil percentage (%)	76.3 ± 7.3	83.3 ± 6.3	86.4 ± 8.2	62.4 ± 10.1
	Lymphocyte percentage (%)	14.0 ± 5.5	9.8 ± 5.1	7.8 ± 5.0	27.8 ± 9.4
	Hematocrit	0.30 ± 0.039	0.27 ± 0.045	0.28 ± 0.059	0.43 ± 0.030
	Platelet (×10^9^/L)	276 ± 86	207 ± 92	186 ± 139	257 ± 70.9
Liver function	Total protein (g/L)	55.7 ± 6.6	54.1 ± 6.9	51.6 ± 6.2	74.1 ± 4.9
	Albumin (g/L)	32.8 ± 3.4	30.9 ± 3.3	29.6 ± 2.8	47.1 ± 2.2
	Total bilirubin (μmol/L)	9.8 ± 5.1	16.4 ± 17.2	40.5 ± 8.7	11.9 ± 2.8
	Direct bilirubin (μmol/L)	5.0 ± 2.6	9.5 ± 14.0	29.9 ± 9.0	4.1 ± 1.2
	Indirect bilirubin (μmol/L)	4.8 ± 2.8	6.9 ± 4.3	10.6 ± 6.8	7.8 ± 1.9
Renal function	Creatinine (μmol/L)	58 ± 50	103 ± 133	136 ± 120	72 ± 21
	Urea N (mmol/L)	7.4 ± 12.1	6.1 ± 5.0	8.9 ± 7.0	5.8 ± 1.4
	Uric acid (μmol/L)	203 ± 105	166 ± 95	219 ± 134	299 ± 110
Blood coagulation	Prothrombin time (s)	13.0 ± 0.9	15.6 ± 6.1	16.4 ± 4.6	12.4 ± 1.0
	Partial thromboplastin time (s)	37.3 ± 7.2	53.9 ± 31.3	59.6 ± 28.9	28.8 ± 2.3
	International normalized ratio	1.14 ± 0.080	1.36 ± 0.54	1.43 ± 0.41	1.08 ± 0.089
	Fibrinogen (mg/dL)	4.44 ± 0.81	4.21 ± 1.18	4.10 ± 1.09	2.63 ± 0.64
Infection	Microbiologically confirmed (%)	0	25.0	35.3	0
	Clinically proven or suspected (%)	0	100.0	100.0	0
Type of organisms	Gram-positive (%)	0	6.3	5.9	0
	Gram-negative (%)	0	18.7	23.5	0
	Fungus (%)	0	0	5.9	0

*N/A, no available*.

### Sampling and neutrophil isolation

Peripheral blood was sterilely collected at the days in which sepsis was definitely diagnosed and immediately delivered to our laboratory. The sample was then divided into 3 aliquots of 200 μL in the biosafety cabinet and stored at −80°C. Another portion (2 mL) of each sample was immediately used for isolation of neutrophils with commercially Ficoll-dextran reagents. Neutrophil-rich pellets were subjected to hypotonic lysis of the remaining erythrocytes with E-lysis. Cell pellets were resuspended in DMEM supplemented with 10% FCS (heat inactivated). Cells were incubated in polypropylene tubes (Falcon/Becton Dickinson, Cambridge, UK) to prevent adherence. The purity of neutrophils was >95%, as assessed by CD16^+^ cell by flow cytometry. Isolated neutrophils were stored at −80°C until DNA extraction and proteomics analysis.

### DNA extraction and polymerase chain reaction (PCR)

DNA from whole blood and isolated neutrophils was extracted with the QIAamp DNA Mini Kit (Qiagen, Valencia, CA) following the manufacturer's instructions. For extraction of neutrophil DNA, a bead-beating step (FastPrep machine for 45 s at setting 5.5, Bio 101) after the addition of the RLT buffer was done to enhance cell lysis. The hypervariable V3 region of the 16S rRNA gene was amplified using universal primer set 357f (5′-TACGGGAGGCAGCAG-3′) and 518r (5′-ATTACCGCGGCTGCTGG-3′) (Li et al., 2013b). An aliquot of DNA (100 ng) recovered from the blood and neutrophils was added into a reaction mixture, and PCR reactions were carried out with a touchdown thermocycling program. The cycling was as follows: initial denaturation at 94°C for 5 min, 30 s at 94°C (denaturation), 30 s at 65°C (annealing), and 30 s at 68°C (elongation) with a 0.5°C touchdown every second cycle during annealing for 20 cycles, followed by 15 cycles with an annealing temperature of 56°C and a final cycle consisting of 5 min at 68°C. The purity and correct size of the resulting PCR amplicons (approximately 190 bp) were assessed on 1% agarose gels, stained with ethidium bromide (5 μg/mL) and visualized under UV light. To ascertain the specificity of the primers (no eukaryotic, mitochondrial, or Archea DNA targeted), we sequenced the PCR products (~10 ng each sample) by Sanger technologies. The sequencing results showed that only 16S rDNA bacterial fragments were yielded from the amplification, confirming the specificity of the primers.

To ensure absence of false positive amplifications, we conducted real-time quantitative PCR with the primer set (357f/518r) to test for possible bacterial contaminants from the reagents (PCR mixtures, solutions for DNA extraction and sterile water) and consumables. The standard curve for the quantification was performed by generating a series of 10-fold dilutions from 5 × 10^3^ to 5 × 10^8^ of 16S rRNA gene copies per reaction using plasmid DNA containing the complete 16S rRNA gene sequence of an *E. coli* DH5α strain. Amplifications of samples and standard dilutions were performed in duplicates on the AB7500 real time PCR system (Life Technologies, CA). The quality of the amplifications was assessed by melting curves. The data showed that the background signal, represented by negative controls (NC), was far lower than those of blood samples (Supplementary Figure 1), indicating the absence of bacterial DNA contaminants from reagents and consumables.

### DNA library construction and 16S rRNA gene sequencing

PCR products were purified with Agencourt AMPure beads (BeckmanCoulter). An aliquot (50 ng) of purified DNA was used for construction of barcoded libraries using the Ion Plus Fragment Library Kit (Life Technologies). In this step, a sample-specific “DNA molecular tag (barcode),” a 14-base semirandom sequence, was intended to uniquely identify original template molecules. DNA concentrations of the libraries were estimated with a Qubit dsDNA HS kit. Libraries for each run were diluted to 26 pM for template preparation. Emulsion PCR was carried out using the Ion OneTouchTM 200 Template Kit v2 (Life Technologies). Sequencing of amplicon libraries was conducted on 316 v2 chips using the Ion Torrent PGM system with the Ion Sequencing 200 kit (Life Technologies). After sequencing, the individual sequence reads were filtered by the PGM software to remove low quality and polyclonal sequences. All PGM quality-approved, trimmed and filtered data were exported as FASTQ files.

The sequence data were deposited in the NCBI Bioproject and the Sequence Read Archive with accession codes PRJNA428535 and SRP128069, respectively.

### Sequence processing and quality control

Sequenced files were online converted to FASTA format and were filtered to remove low-quality sequences with the Galaxy projects (https://usegalaxy.org/). A mean quality score of ≥20 and a minimum length of 150 bp for the coupled V3 region were required. The resulting sequences were then aligned online to operational taxonomic units (OTUs) (97% identity) with the CD-HIT (http://weizhong-lab.ucsd.edu/cdhit_454/cgi-bin/index.cgi) (Li and Godzik, 2006). Sequences that did not match the defined core region of the seed alignment were manually removed. OTUs were classified taxonomically to bacterial genera using the Ribosomal Database Project (RDP) classifier with a 50% bootstrap threshold (http://rdp.cme.msu.edu/classifier/classifier.jsp) (Wang et al., 2007).

### Protein extraction, digestion, and iTRAQ labeling

Isolated neutrophils were thawed and resuspended in lysis buffer of cold acetone containing 10% trichloroacetic acid (TCA) and 10 mM dithiothreitol (DTT) and sonicated for 3 times on ice to enhance cell lysis. The protein pellets were then collected by centrifuging and resuspended in a buffer (7 M urea, 2 M thiourea, 4% CHAPS, 30 mM, Tris–HCl pH 8.0), containing 1 mM phenylmethylsulfonyl fluoride (PMSF), 2 mM ethylenediaminetetraacetic acid (EDTA) and 10 mM DTT. Samples were again sonicated and centrifuged and subsequently, the supernatant was reduced and alkylated by 10 mM DTT and 55 mM iodoacetamide (IAA). The treated proteins were precipitated with chilled acetone (1:4) at −20°C overnight. The precipitants were resuspended in 500 mM triethylammonium bicarbonate (TEAB), then sonicated and centrifuged as above. The protein content of the supernatant was determined using the Bradford method. The resulting proteins (~100 μg) of each sample were digested by and then labeled with 6-plex iTRAQ reagents containing 6 different stable-isotope (126–131) covalent mass tags (Applied Biosystems) according to the manufacturer's protocol.

### Peptide fractionation and mass spectrometry (MS) analysis

The labeled peptides were pooled, eluted and resolved into 10 fractions using an Ultremex SCX column containing 5-μm particles (Phenomenex, USA). The eluted fractions were desalted using a Strata X C18 column (Phenomenex, USA) and dried under vacuum. Each fraction was resuspended in a certain volume of buffer A (2% acetonitrile, 0.1% formic acid, pH3.0) and centrifuged at 20,000 × g for 10 min. The final concentration of peptide was about 0.5 μg/μl on average in each fraction. Supernatant was loaded on a Nano ACQUITY UPLC system using the autosampler. The peptides were subjected to nanoelectrospray ionization followed by tandem mass spectrometry (MS/MS) in a LTQ-Orbitrap (Thermo Fisher Science, USA) coupled online to the HPLC. Intact peptides were detected in the Orbitrap at a resolution of 60,000. Peptides were selected for MS/MS using high energy collision dissociation (HCD) operating mode with a normalized collision energy setting of 45%. LC-MS/MS was operated in positive ion mode as described. The analytical condition was set at a linear gradient from 0 to 60% of buffer B (CH_3_CN) in 150 min, and a flow rate of 200 nL/min. Ion fragments were detected on the LTQ. A data-dependent procedure that alternated between one MS scan followed by eight MS/MS scans was applied for the eight most abundant precursor ions above a threshold ion count of 5,000 in the MS survey scan. The electrospray voltage applied was 1,500 V. Automatic gain control (AGC) was used to prevent overfilling of the ion trap; 1 × 10^4^ ions were accumulated in the ion trap for generation of HCD spectra. For MS scans, the m/z scan range was 350 to 2,000 Da.

### MS data processing, protein quantization, and functional annotation

The MS/MS spectra acquired from precursor ions were submitted to Maxquant (version 1.2.2.5) using the following search parameters: the database for the search was Uniprot proteome (version 20140418); the enzyme was trypsin (full cleavage); dimethylation labeling for quantification; the dynamic modifications were set for oxidized Met (+16); carbamidomethylation of cysteine was set as static modification; MS/MS tolerance was set at 10 ppm; the minimum peptide length was 6; the false detection rates for both peptides and proteins were all set below 0.01. All identified peptides had an ion score above the identity threshold, and a protein was considered identified if at least one such unique peptide match was apparent for the protein. Individual quantitative samples were normalized within each acquisition run according to the algorithm described in i-Tracker (Shadforth et al., 2005). The logic algorithm for set operations was applied to further screen for differentially expressed proteins identified in the present study. Gene Ontology (GO) functional annotation was carried out using Blast2GO software (Conesa et al., 2005).

### Flow cytometry

Blood specimens were freshly collected for assessment of lymphocyte subsets, cell apoptosis, and HLA-DR expression on monocytes. For measurement of cell apoptosis, mononuclear cells were prepared by density gradient centrifugation with Ficoll-Paque (Stem cell Technologies), and were then stained by Annexin V-PE, 7-ADD-PerCP, and fluorescein-labeled mAbs against CD4, or CD8, respectively. Assessment of T lymphocyte subsets, HLA-DR and T-helper (Th) cells was performed using commercial kits (BD Biosciences). After activation with phorbol myristate acetate and ionomycin, immunostaining was performed using fluorescein-labeled mAbs against CD4, interferon-gama (INF-γ), interleukin (IL)-4 and IL-17 (BD Pharmingen). A logical gate combining CD4^+^ cells and their scatter properties was used for the phenotypes of Th1, Th2, and Th17 cells. The proportions of the peripheral naïve CD4 T cells (CD3^+^CD4^+^CCR7^+^CD45RA^high^CD28^+^), naïve CD8 T cells (CD3^+^CD8^+^CCR7^+^CD45RA^high^CD28^+^), memory CD4 T cells (CD3^+^CD4^+^CD45RA^−^), memory CD8 T cells (CD3^+^CD8^+^CD45RA^−^), effector memory (CD3^+^CD8^+^CCR7^−^CD45RA^−^), and terminal effector memory (CD3^+^CD8^+^CCR7^−^CD45RA^high^CD28^−^) were also measured, respectively (Qi et al., 2014). All data were acquired on a Becton Dickinson FACSCanto II and analyzed with CellQuest software (BD Biosciences).

### Assay of serum cytokines

Measurement of serum TNF-α, IL-1β, IL-6, IL-10, IL-18, and IFN-γ levels was performed in duplicate with enzyme-linked immunosorbent assay (ELISA) kits (R&D Systems, Abingdon, UK).

### Statistical analysis

Quantitative data are represented as means ± standard deviation (SD). Statistical analysis was performed by one-way analysis of variance (ANOVA) followed by the Holm-Sidak test using SPSS software (version 12.0). A *P* < 0.05 was considered significant. The species richness in the bacterial microbiota was estimated by the OTU numbers at the same sequencing depth, which was compared to reflect the difference of the microbiota diversity between groups. Correlation between two variances was estimated using linear regression analysis with a Pearson's test in R software (http://www.r-project.org/). Heatmaps were generated for non-scaled, non-normalized titer data using a Euclidean distance function with complete linkage clustering or non-clustering in R using the package pheatmap (version 3.1.1). Principal component analysis was conducted with Canoco software for Windows 4.5 (Microcomputer Power, Ithaca, NY). The output matrix containing the relative abundance of OTUs per sample was processed with the linear discriminant analysis effect size (LEfSe) algorithm (Segata et al., 2011) using an alpha cutoff of 0.05 and an effect size cutoff of 2.0.

## Results

### Characterization of bacterial microbiomes in peripheral blood of SAP patients

To characterize the bacterial communities possibly present in systemic circulation, we sequenced 16S rRNA gene amplicons of blood samples from 50 patients with SAP and 12 healthy subjects. The patients were classified into three groups: uninfected (*n* = 17), infected (*n* = 16), and septic (*n* = 17) (Table 1 and Supplementary Table 1). Prior to sequencing, we conducted a quantitative polymerase chain reaction (qPCR) assay to determine the concentration of 16S rRNA gene copies in the whole blood of each subject (Supplementary Figure 1). The numbers of 16S rDNA copies in blood were significantly higher in the infected and septic patients than in healthy controls (on average 1.38 × 10^8^ copies per milliliter) (*P* < 0.01) (Supplementary Figure 1D). After sequencing the 16S rDNA amplicons, we generated over 80,000 sequences for each sample, and unique sequences were clustered into 14,526 OTUs (97% ID). Taxonomic classification showed that these OTUs were assigned into 335 bacterial genera, mainly affiliated with the four major phyla: Proteobacteria, Bacteroidetes, Firmicutes and Actinobacteria (Supplementary Figure 2A). An average of 204 OTUs (range: 50–694) were obtained from the patients' samples (Supplementary Figure 2B), indicating that a diverse bacterial microbiome, rather than one or several bacterial species as thought previously, was present in peripheral circulation of the patients. Surprisingly, we also observed a highly diverse microbiota in the blood of healthy subjects (on average 360 OTUs, range: 265–520) (Supplementary Figure 2B). As compared to healthy controls, the species richness of the blood microbiota, estimated by the number of OTUs (at the same sequencing depth), was significantly reduced in SAP patients (*P* < 0.05) (Supplementary Figure 2B). Additionally, the composition of the microbiota was profoundly distinct between the patients and healthy controls, characterized by severe depletion of Actinobacteria (*P* < 0.0001) and an overgrowth of Bacteroidetes in the former (*P* < 0.05) (Supplementary Figure 2C). At the class level, we saw a marked increase in Bacteroidia and Clostridia, and a striking reduction in Actinobacteriae, Flavobacteriia and Bacilli in the patients vs. healthy controls (*P* < 0.05) (Supplementary Figure 2C). Principal component analysis (PCA) of the weighted UniFrac distances, based on the data of genus-level relative abundance, showed a clear separation of the patients' samples from healthy controls along PC1 and PC2, indicating the differences of the microbiota profiles between them (Supplementary Figure 2D). However, no significant difference was found in the microbiota structures across the patient groups, as scattered distribution of their sample dots in the PCA plot.

### Potential source of the blood microbiota in SAP patients

**Figure 1:**
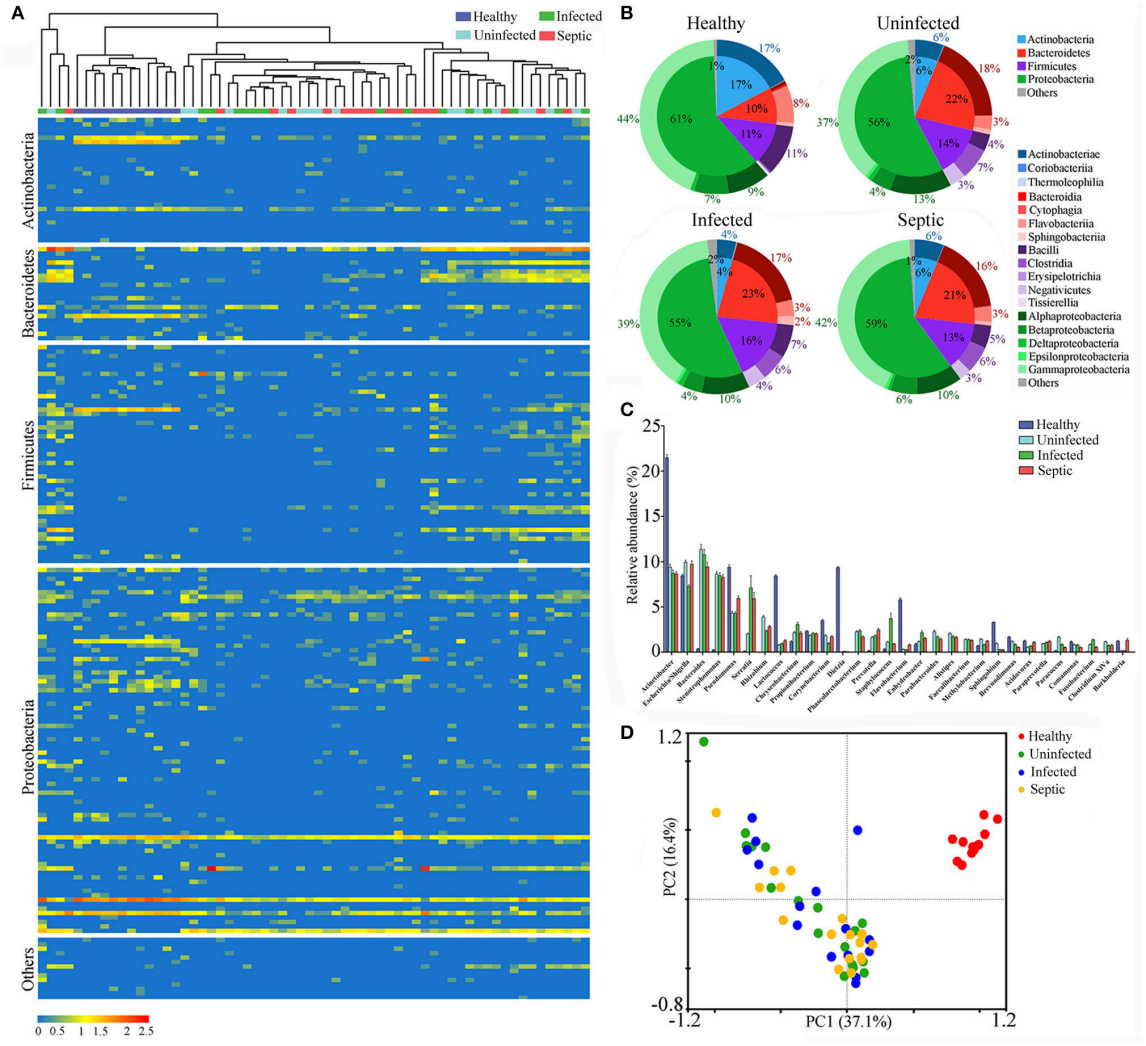
Characterization of the blood bacterial microbiomes potentially derived from the gut. **(A)** Heatmap showing the abundance of the putative gut-derived bacterial genera identified by taxonomic classification. The data represent the Log10 values of the operational taxonomic unit (OTU) counts each genus. The clustering relationships across the blood samples are shown in the upper panel. **(B)** The pie charts indicating the composition of the putative gut-derived organisms in the blood microbiota at the phylum and class levels. **(C)** Comparisons in the relative proportions of the top 30 bacterial genera among groups. **(D)** The plot of principal component analysis (PCA) showing the difference of the microbial community structures.

To track the possible source of blood microbiomes, we compared our sequences to the 16S rRNA gene dataset from the National Center for Biotechnology Information (NCBI) (The Human Microbiome Project Consortium, 2012). Consequently, an average of 87.0% (range: 73.5–98.3%) of the blood microbiome memberships across individuals were taxonomically classified as known commensal or pathogenic bacteria colonizing the human gut, far higher than those from other sites (Supplementary Figure 3A). Given the dominance of the putative gut-derived organisms within the niches, we estimated their contribution to the microbiome-wide alterations in SAP patients (Figure 1A). The counts of the OTUs likely affiliated with the gut organisms was markedly declined in patients relative to the healthy controls (*P* < 0.05) (Supplementary Figure 3B). Comparison of phylum-level proportions indicated pronounced variations of the putative gut-derived organisms in patients, displaying similar patterns vs. the microbiome-wide changes (Figure 1B and Supplementary Figure 2C). The Bacteroidetes was overrepresented while Actinobacteria was markedly decreased in all patient groups (*P* < 0.05) (Figure 1B), followed by a striking increase in the ratios of the relative abundance between both phyla compared to those of healthy subjects (Supplementary Figure 4A). The ratios between Firmicutes and Actinobacteria rose (Supplementary Figure 4B), which was mainly due to significant decline in Actinobacteria in the blood of the patients. Such changes in the predominant phyla provided compelling evidence indicating that the putative gut-derived bacterial composition was significantly altered in patients (Supplementary Figure 3C). Class-level analyses showed highly consistent changes with those of the aggregate microbiota (Figure 1B and Supplementary Figure 2C), suggesting that shifts of the putative gut-derived organisms contributed predominantly to the blood microbiome-wide alterations in patients. At the genus level, 20 of the putative gut-derived bacterial genera, including *Bacteroides* (10.5 ± 9.2%), *Escherichia/Shigella* (9.0 ± 4.6%), *Acinetobacter* (8.9 ± 4.9%), *Stenotrophomonas* (also likely derived from soils) (8.5 ± 4.8%), *Serratia* (4.9 ± 2.8%), *Pseudomonas* (also likely as soil-derived) (4.5 ± 2.8%), *Rhizobium* (also likely as soil-derived) (3.0 ± 2.4%), *Prevotella* (1.9 ± 2.1%), *Corynebacterium* (also likely derived from skin or soils) (1.5 ± 1.7%) and so on, dominated the blood microbiota in patients (average relative abundance >1%) (Figure 1C). These bacterial genera were also abundant in healthy controls; however, their proportions were significantly distinct from those of patients (Figure 1C). The genera *Bacteroides, Stenotrophomonas, Serratia, Rhizobium, Prevotella, Staphylococcus*, and *Paracoccus* were markedly expanded in the blood of the patients, regardless of the illness severities (*P* < 0.05, vs. healthy controls) (Figure 1C). Interestingly, some bacterial genera, such as *Rhizobium, Phascolarctobacterium, Alistipes, Parabacteroides, Faecalibacterium, Paraprevotella*, and *Clostridium XlVa*, were present in the patients, but not detected in healthy subjects. In contrast, the genera *Acinetobacter, Lactococcus, Dietzia, Flavobacterium, Pseudomonas, Corynebacterium, Sphingobium*, and *Brevundimonas*, were consistently decreased in the patients (*P* < 0.05, vs. healthy controls). Of them, some bacterial genera were commonly considered to be potentially pathogenic or probiotic, and were probably of special importance for the blood microbiome alterations in patients (Supplementary Figure 5). Like the blood microbiota-wide analysis, the sample dots, representing the putative gut-derived bacterial communities, displayed highly similar distributions in the PCA plots (Figure 1D and Supplementary Figure 2D), indicating that altered abundance of putative gut-originated organisms was predominantly responsible for the microbiome-wide alteration in patients. To further identify taxonomic differences in the microbiomes between the patients and healthy subjects, we conducted a linear discriminant analysis (LDA) effect size (LEfSe) algorithm. Consistent with the findings mentioned above (Figure 1B), the changes of the blood microbiome in patients were primarily sourced from the classes Bacteroidia, Clostridia, Actinobacteriae, Flavobacteriia and Bacilli (Supplementary Figure 6).

### Identification of diverse bacterial microbiome within neutrophils

To define the configurations of neutrophil-associated microbiomes, also termed as NAMs, we isolated peripheral neutrophils from the patients and healthy subjects, and sequenced the 16S rDNA amplicons obtained from the cells. We observed that a surprisingly diverse microbiome was present within the neutrophils both in patients and in healthy subjects, as indicated by several hundreds to thousands of unique OTUs. Similar to the blood microbiomes, the OTUs obtained from the neutrophil specimens were mainly classified into the four phyla: Proteobacteria, Bacteroidetes, Firmicutes and Actinobacteria (Supplementary Figure 7A). A notable expansion of OTUs numbers was seen in the infected or septic patients (*P* < 0.01) (Supplementary Figure 7B), implying that the neutrophils in these cases might capture more diverse bacteria than in healthy controls. Viewing the microbiota profile, we found that the increase of species richness in infected or septic patients was mainly due to over-presence of certain bacteria belonging to the phyla Bacteroidetes and Firmicutes (*P* < 0.05, vs. healthy controls) (Supplementary Figure 7C). The proportions of the classes Bacteroidia, Clostridia, and Negativicutes were significantly expanded in the patients, whilst Actinobacteriae, Flavobacteriia, Gammaproteobacteria and Betaproteobacteria were markedly declined (*P* < 0.05, vs. healthy controls) (Supplementary Figure 7C). The PCA plots showed that the majority of the patients' sample dots, were distant from those of the healthy controls, suggesting that the NAM shifted toward aberrant configuration in patients (Supplementary Figure 7D).

**Figure 2:**
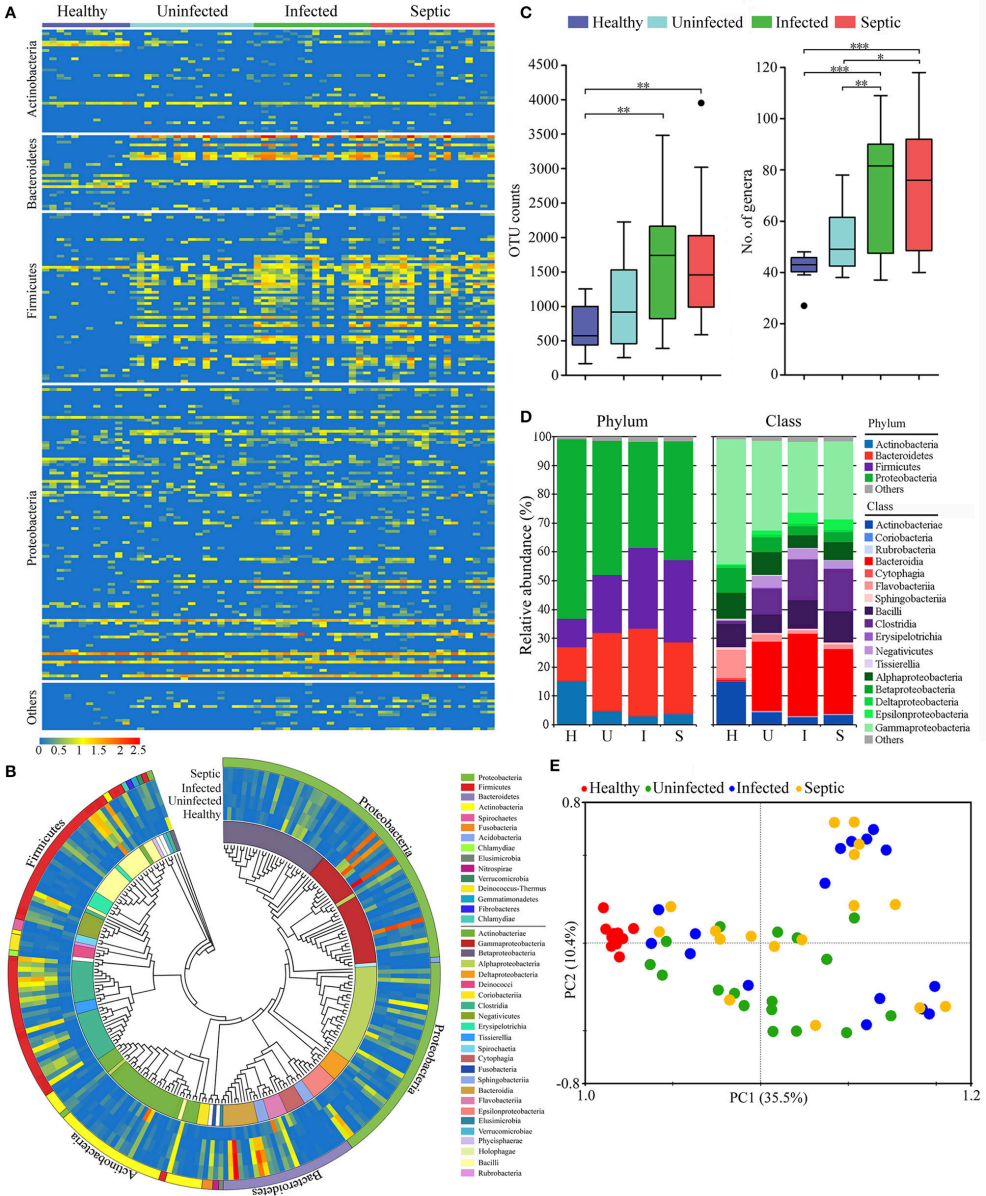
Phylogenetic compositions of putative gut-derived organisms in the neutrophil-associated microbiomes (NAMs). **(A)** Heatmap exhibiting the composition of the bacterial genera likely originated from the gut in peripheral neutrophils. **(B)** The phylogenetic analysis of the putative gut-derived bacterial composition of the NAMs. The data presented in the heatmap represent the means of the Log10 values of the operational taxonomic unit (OTU) counts from each group. **(C)** Differences of the counts the OTUs and genera affiliated with the putative gut-derived organisms in the NAMs. ^*^P < 0.05; ^**^P < 0.01; ^***^P < 0.001. **(D)** Changes of the predominant bacterial composition in the NAMs at the phylum and class levels. The letters “H,” “U,” “I,” and “S” represent the healthy, uninfected, infected and septic groups, respectively. **(E)** Principal component analysis (PCA) of weighted UniFrac distances, based on the relative abundance of each genus, displaying the compositional differences of the NAMs.

Next we determined the potential origin of the neutrophil-associated microbiome, obtaining similar results with that of the blood microbiota. The organisms that presumably derived from the gut constituted the major component of the neutrophil-associated microbiome, with high abundance proportions (on average 83.1% of the aggregate, range: 63.7 to 94.8%) across all samples (Supplementary Figure 8A). The putative gut-derived organisms in neutrophils were profoundly diverse (Figures 2A,B), and especially in infected and septic patients, the counts of the OTUs and bacterial genera were far more than in healthy controls (*P* < 0.01) (Figure 2C). As compared to healthy controls, the most significant shifts of the bacterial communities in neutrophils of patients were the increases in the Bacteroidetes and Firmicutes, together with a profound reduction in Actinobacteria and Proteobacteria (*P* < 0.01) (Figure 2D). The ratios between Bacteroidetes or Firmicutes and Actinobacteria were significantly higher in the patients than those of healthy microbiotas (*P* < 0.01) (Supplementary Figure 8B). At the class level, the bacterial microbiomes in neutrophils differed markedly between the patients and healthy individuals, mainly characterized by an expansion in Bacteroidia, Clostridia and Negativicutes, as well as a reduction in Actinobacteriae, Flavobacteriia, Gammaproteobacteria, and Betaproteobacteria (*P* < 0.01) (Figure 2D). The proper proportions of such bacterial taxons appeared to be disrupted, indicating significant alterations in the neutrophil-associated microbiomes of the patients. The PCA plot, based on the relative abundance of the putative gut- derived bacterial genera, indicated that the microbiome memberships were distinguished between healthy controls and patients (Figure 2E). To explore the bacterial phylotypes associated with the shifts of the neutrophil-associated microbiomes in the patients, we compared the relative abundance of some specific bacterial genera that were often included in the potentially pathogenic and probiotic organisms (Supplementary Figure 9A). Of them, the potentially pathogenic organisms, including *Bacteroides, Stenotrophomonas, Clostridium* XIVa, *Fusobacterium, Eubacterium*, and *Serratia* were more enriched in the patients than healthy individuals (*P* < 0.05) (Supplementary Figure 9B). However, the genera *Acinetobacter, Lactococcus, Corynebacterium, Flavobacterium, Pseudomonas, Bifidobacterium, Legionella*, and *Anaerococcus* were significantly less abundant in the patients (*P* < 0.05, vs. Healthy controls) (Supplementary Figure 9B). Further, we conducted the linear discriminant analysis (LDA) effect size (LEfSe), indicating the variations of the neutrophil-associated microbiomes in patients (Supplementary Figure 10). In total, the neutrophil-associated microbiome in the patients was distinct from that of healthy subjects, which probably had important implications in the development of bacteremia and systemic infection.

### Associations between immune traits and blood microbiome in SAP patients

**Figure 3:**
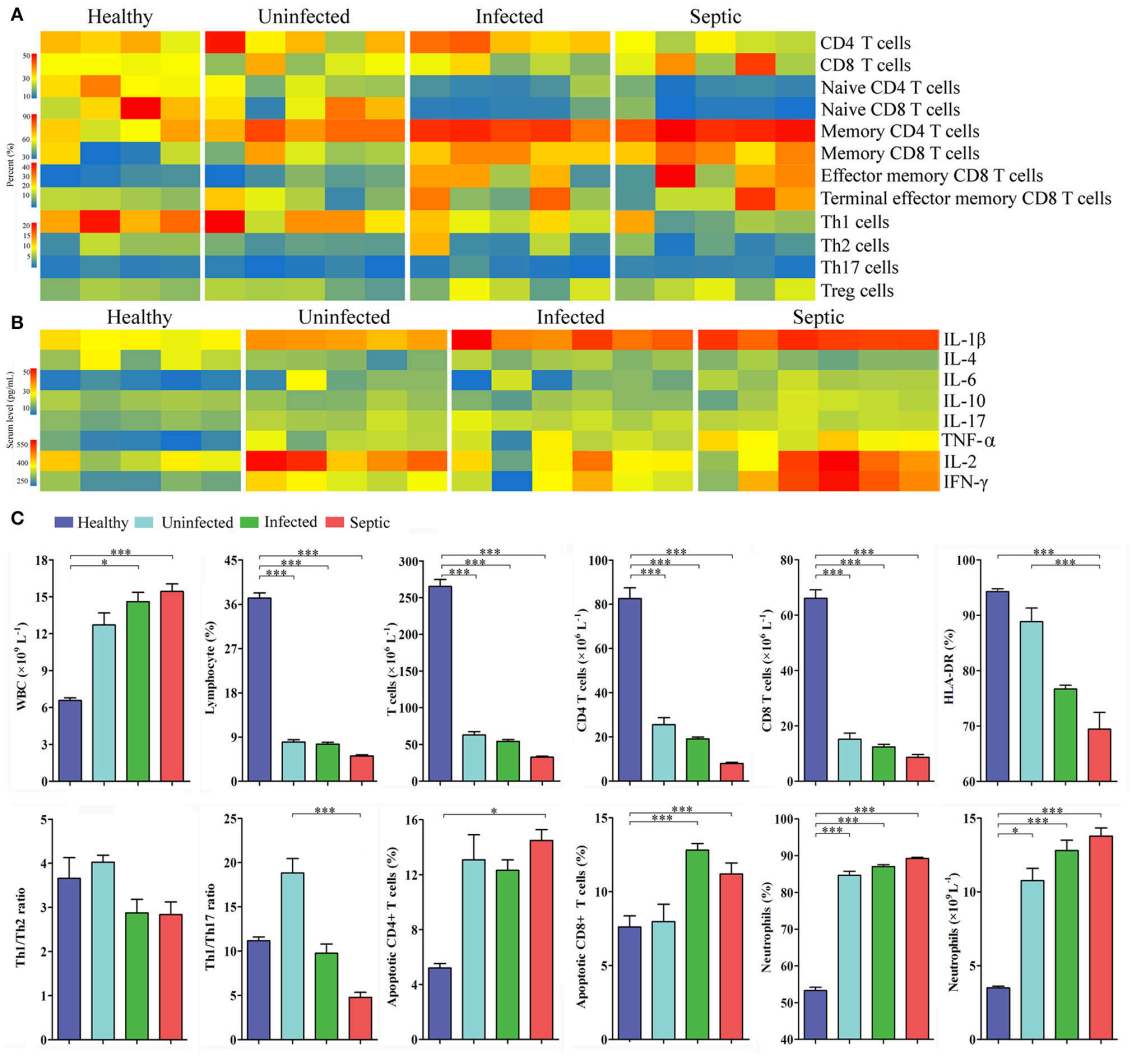
Changes in the peripheral immune cell subpopulations and serum cytokines. **(A)** Heatmap displaying the variations of the lymphocyte subsets in SAP patients compared to healthy controls. **(B)** Profiles of the serum cytokines in patients and healthy controls. **(C)** The graphs indicate the changes of T-helper cell subsets, apoptotic lymphocytes and neutrophils, together with the counts of peripheral immune cells in patients. ^*^P < 0.05; ^***^P < 0.001.

Next we characterized a wide range of circulating immune cell subtypes in the peripheral blood samples from the patients and healthy controls. As shown in the Figure 3A, the percentages of CD4^+^ and CD8^+^ T lymphocyte subsets were both reduced in the septic patients (*P* < 0.01, vs. healthy controls). The proportions of naïve T cells (CD4^+^, CD8^+^) were also decreased strikingly in the infected and septic cases, whilst the memory T cells were increased compared to those of healthy subjects (*P* < 0.01). In addition, the counts of the total lymphocytes, CD4^+^ and CD8^+^ T lymphocytes were significantly decreased in the patients (*P* < 0.01, vs. healthy controls), reaching the minimum values in septic cases (Figure 3C). By contrast, a significant increase in CD8^+^ T cell apoptosis was observed in the infected and septic patients (*P* < 0.001) (Figure 3B). The apoptosis of CD4^+^ T cells was also increased in uninfected, infected and septic cases, while a significantly statistical difference was only found between septic patients and healthy controls (*P* < 0.05). Of special note, the septic patients displayed a strong proinflammatory cytokine profile, characterized by increased release of IL-1β, IL-2, IL-6, TNF-α, and IFN-γ in the serum (*P* < 0.001) (Figure 3B).

**Figure 4:**
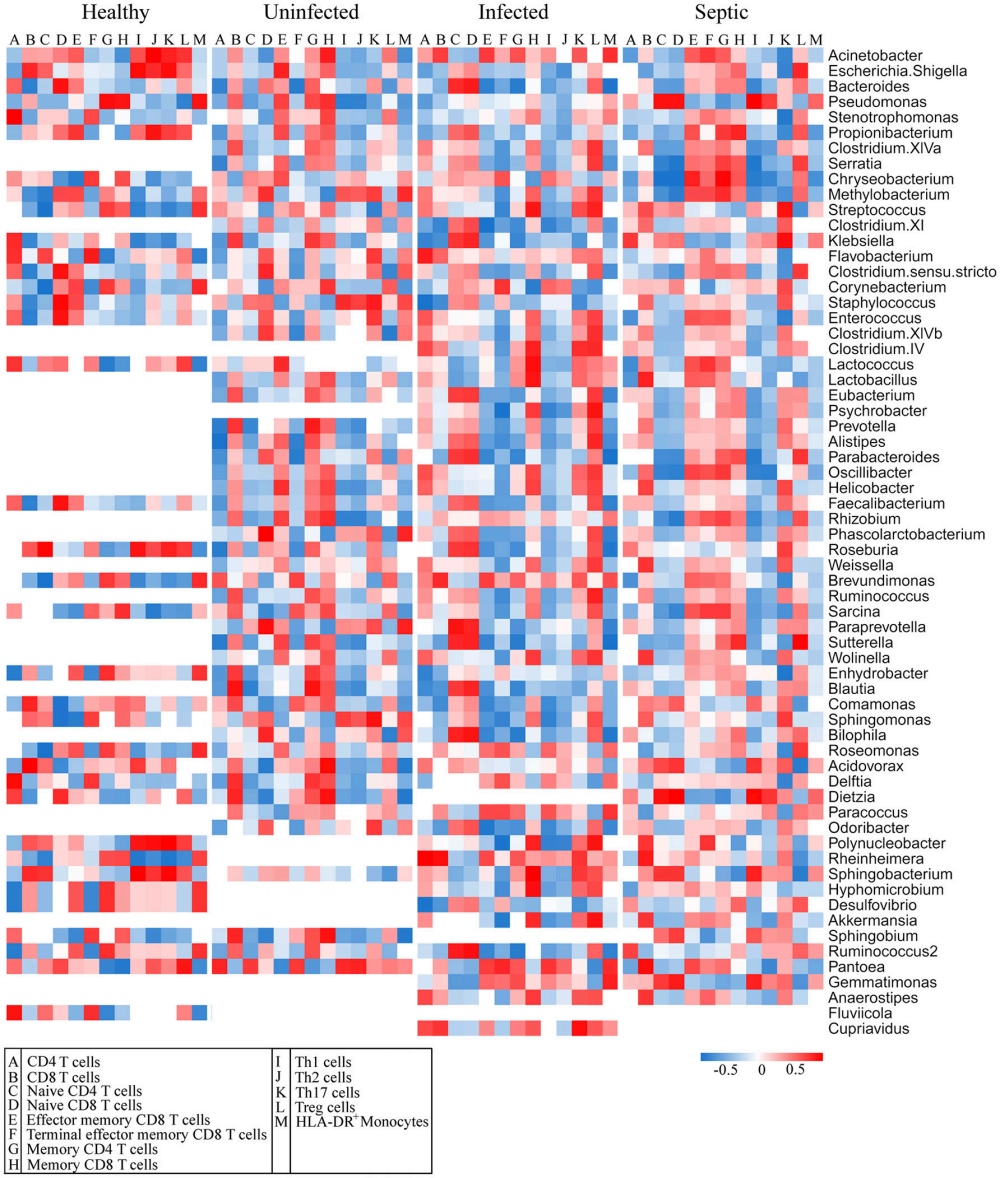
Correlations of the predominant bacterial genera in the neutrophils with the subpopulations of peripheral lymphocytes. The data presented in the heatmap indicate the correlation coefficient between variances.

The blood and neutrophil-associated microbiomes of the patients were distinctfrom those of healthy subjects, raising a question of whether shifts of the microbiomes were related to the immunological disorders in patients. To address it, we examined relationships between specific clades of the microbiomes and the immunological parameters in patients (Figure 4). We observed that some bacterial genera associated with the neutrophils, such as *Acinetobacter, Bacteriodes, Stenotrophomonas, Serratia, Pseudomonas, Chryseobacterium, Methylobacterium, Clostridium, Enterococcus, Lactococcus*, and *Oscillibacter*, etc., were correlated either positively or negatively with T lymphocyte subsets, especially naïve and memory T cells, in the septic patients (Figure 4). Similarly, some of the bacterial phylotypes showed strong correlation with the immunological traits in the uninfected and infected patients. The majority of these bacteria in the neutrophil-associated microbiomes were closely associated with the changes of serum cytokine levels in patients (Supplementary Figure 11). However, the bacterial genera that closely correlated to the lymphocyte subsets and serum cytokine concentrations were relatively fewer in healthy controls. In addition, some bacterial taxa in the peripheral blood were significantly correlative with the immunological parameters both in patients and in healthy subjects (Supplementary Figures 12, 13). Clearly, the changes of the blood and neutrophil-associated microbiomes were strongly linked to immunological disorders of patients.

### Changes of neutrophil proteomic profiles and its connection with alterations of blood microbiome

**Figure 5:**
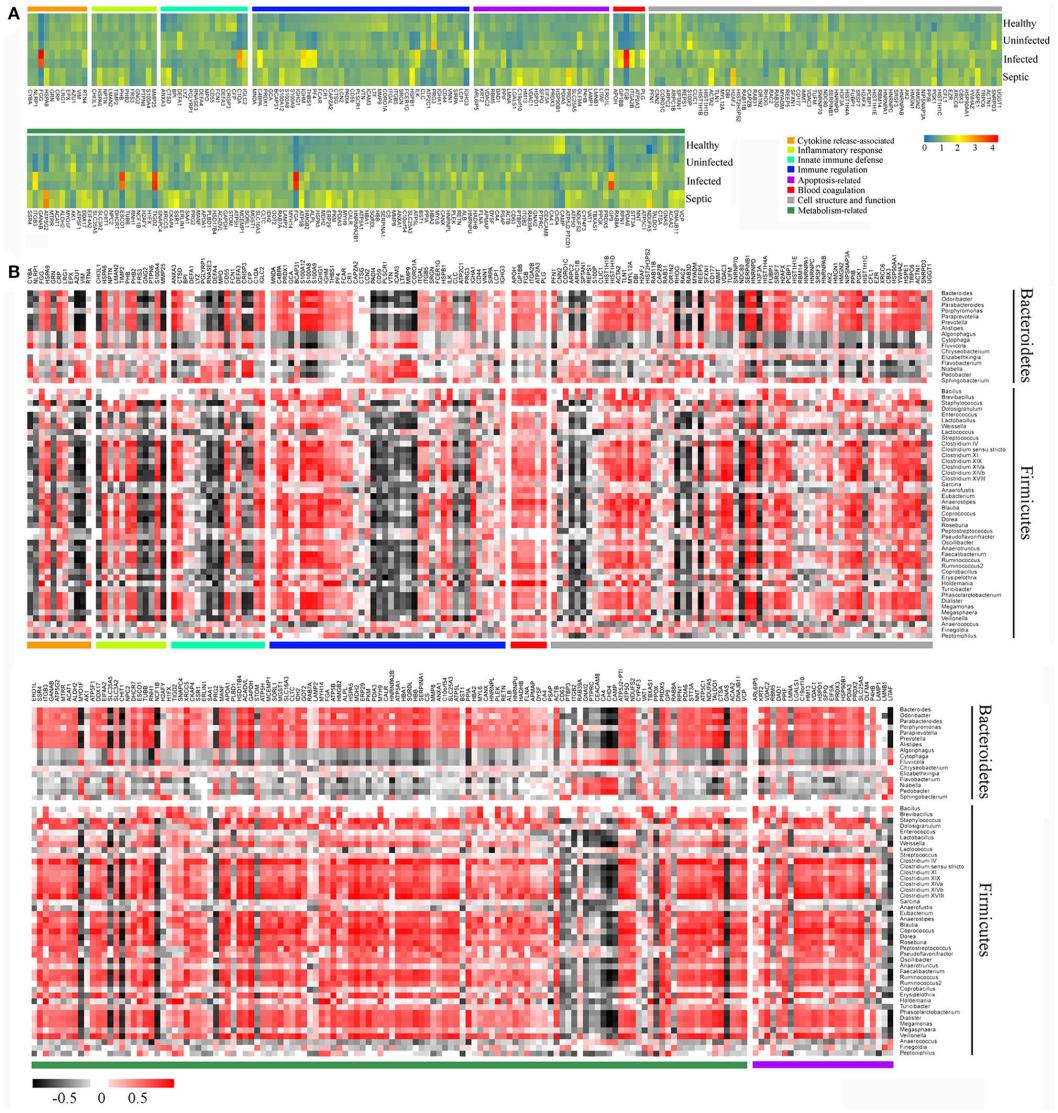
Association between alterations of neutrophil proteomics profiling and dysbiotic microbiota**. (A)** Heatmap showing the changes of the relative abundance of expressed-differentially proteins in neutrophils. **(B)** Correlations between the expressed-differentially proteins of neutrophils and the predominant bacterial genera from the neutrophil-associated microbiomes. The data indicate the correlation coefficient between variances.

We further performed comparative proteomic analysis on peripheral neutrophils derived from healthy and SAP subjects in an attempt to link cell functional changes to the microbiome alterations. A total of 296 proteins were characterized as differentially expressed, were then annotated and clustered into six categories involved in biological functions of neutrophils, including innate immune defense, immune response, cytokine release, cell apoptosis, cell structure, and metabolic activity (Supplementary Table 2). As shown in the Figure 5A, the expression profiles of these proteins were dramatically varied in SAP patients with sepsis and healthy controls. Of these, the proteins closely involved in bactericidal activities of neutrophil, such as lysozyme C (LYZ), eosinophil cationic protein (RNASE3), myeloperoxidase (MPO), neutrophil defensin 3 (DEFA4), properdin (CFP) and bactericidal permeability-increasing protein (BPI), were significantly down-regulated in septic patients, indicating that the dysfunction of innate immunity might be present in septic cases. We also compared the expression profiles of some known immune response-associated proteins, as characterized by significant down-regulations for a vast majority of these proteins in SAP patients (Figure 5A). The changes in the protein expressions were likely, at least in part, involved in the immunosuppression in SAP patients with sepsis. Of special note, matrix metallopeptidase 9 (MMP9), a protein prompting leukocyte transendothelial migration, was significantly declined in septic patients, suggesting the presence of neutrophil dysfunction. The expression of the proteins involved in modulation of proinflammatory cytokines was strikingly up-regulated in septic patients, probably having implication for establishment of the uncontrollable inflammatory response. Similarly, the expression of some proteins associated with cell apoptosis was increased in septic patients, which may help to explain delayed neutrophil apoptosis in sepsis. In addition, an over-representation in metabolism-related proteins was observed in the septic patients, when compared against the data set from healthy persons. Basing on the observations, we provide indirect evidence indicating that the function of neutrophils might be impaired in SAP patients with sepsis.

Next we conducted a Pearson's correlation analysis to associate the differentially expressed proteins of neutrophils with microbiome memberships (Figure 5B). Our data showed that changes of the protein expression in neutrophils were closely associated with specific bacterial taxons of the microbiome. As shown in Figure 5B, most of the proteins in the functional clusters of innate immune defense, immune response and cytokine production correlated negatively with compositional changes of the neutrophil-associated microbiome, especially the memberships of the phyla Bacteroidetes and Firmicutes. On the contrary, a majority of the proteins involved in the apoptosis and metabolic activities of neutrophils correlated positively with the members of the microbiomes.

## Discussion

To our knowledge, this is the first prospective observational case series exploring the microbial landscape in peripheral blood and neutrophils in SAP patients, as well as their potential links with the immunological disorders of the patients. Through this study, we identified diverse bacterial microbiomes within peripheral blood and neutrophils, and discovered that the microbiome is altered in SAP patients. More importantly, the abundance changes in certain members of the microbiomes are closely linked to the immunological disorders of the patients. Our findings provide emerging evidence supporting the presence of the blood microbiome and give us novel insights into induction of bacteremia in SAP patients.

Recent studies have begun to document that human blood contains an authentic microbiome, which could contribute significantly to the development of sepsis (Grumaz et al., 2016; Gosiewski et al., 2017) and several chronic diseases (Amar et al., 2013; Rajendhran et al., 2013; Dinakaran et al., 2014). Yet, whether a diverse bacterial community is present in blood of SAP patients remains an unanswered question. Through the fingerprinting approach of 16S rRNA amplicons, we have recently demonstrated that multiple bacterial species are commonly seen in blood of patients with SAP (Li et al., 2013a), but without deep sequencing data it was not possible to define the microbial landscape in blood and its potential role in pathogenesis of sepsis. Herein we conduct 16S rRNA gene sequencing to characterize the bacterial profile present in blood of SAP patients. We show that the peripheral blood in SAP patients has a diverse bacterial microbiota, dominated by the phyla Proteobacteria, Actinobacteria, Bacteroidetes, and Firmicutes, which is consistent with previous reports (Amar et al., 2013; Rajendhran et al., 2013; Dinakaran et al., 2014). Furthermore, the blood microbiome in SAP patients appears dysbiotic, as revealed by loss of species richness and changes of predominant bacterial taxa relative to healthy controls. Together with the findings, we suggest that the perturbation of blood microbiota, which likely represents a disease-provoking state, might be involved in the progression of sepsis. Prior studies have reported that dysbiosis of the blood microbiome is an independent risk factor of cardiovascular disease, indicating its potentially pathological role (Amar et al., 2013; Rajendhran et al., 2013; Dinakaran et al., 2014). In total, our investigations based on culture-independent techniques have shown a previously unappreciated complexity of the blood bacterial microbiome in SAP patients, also forcing reconsideration of the mechanisms of sepsis pathogenesis and exacerbations.

Intestinal dysbiosis and bacterial translocation are common in critically ill patients, and there is strong evidence that the translocation of bacteria and their products across the intestinal barrier drives the progression of sepsis (Dickson, 2016; Alverdy and Krezalek, 2017). Our understanding of the concept of translocation of one or several organisms from the gut is founded on culture-based studies (MacFie et al., 1999; MacFie, 2004). Recently, Dickson and colleagues have demonstrated that the lung microbiome is enriched with gut-associated bacteria in sepsis and acute respiratory distress syndrome, providing strong evidence for gut–lung translocation of bacterial microbiota (Dickson et al., 2016). The observations prompted us to re-consider the current opinion of bacterial translocation from the gut to systemic circulation. Our data presented here reveal that the blood microbiome is mainly composed of gut-associated organisms in SAP patients, which is similar to the results in the lung microbiome (Dickson et al., 2016). It is therefore speculated that a bacterial consortium from the gut, rather than single or several organisms, might migrate into systemic circulation during sepsis. A longitudinal study of paired stool and blood specimens will be required to determine the true prevalence for gut-blood translocation of bacterial microbiota in sepsis. Nonetheless, the significant correlation between circulating bacteria and clinical manifestations offers evidence that the blood microbiome may play an important role in sepsis, even in the absence of gut–blood translocation.

Innate immune cells and microorganisms in blood are highly interactive, maintaining a delicate balance between defending against infection and eliciting an excessive inflammatory response (Nathan, 2006). Given the critical role of neutrophils in eradication of pathogens, we sought to characterize the composition of neutrophil-associated microbiomes and explore potential roles in the alteration of blood microbiome in SAP. We show that the neutrophils contain a diverse microbiome, and the microbiota composition is significantly altered in SAP patients, consistent with the findings from the blood microbiome. Proteomic analysis of the neutrophils shows that the function of neutrophils is likely disturbed, especially in septic patients, as revealed by reframing of the protein profiles. The bactericidal effector function of neutrophils, represented by decreased antibacterial peptide production (Flannagan et al., 2009), is probably restricted in SAP patients with sepsis. Another prominent feature of neutrophils is decreased expression of MMP9, possibly contributing to impairment of cell migration during systemic infections (Kolaczkowska et al., 2009). The observations indicate that the neutrophil function is probably collapsed, leading to impaired intracellular bacterial clearance and compositional shifts of NAMs in sepsis. Further investigations with neutrophil killing/phagocytosis assays will be needed to provide direct evidence linking the blood microbiome alterations seen in SAP patients with neutrophil dysfunction.

Unlike other organs, the blood was originally presumed to be sterile, and microbes were thought to be present in circulation only in sepsis cases. But, in recent years, the presence of bacterial 16S rRNA genes has been reported in the circulation of healthy individuals (Potgieter et al., 2015; Païssé et al., 2016). In this study, we have added evidence suggesting that the peripheral blood harbors a diverse bacterial microbiota in healthy individuals, consistent with the previous reports (Païssé et al., 2016). Interestingly, the blood microbiota is enriched with the putative gut-derived bacteria, implying that translocation of intestinal bacteria may play a critical role in shaping the unique microbiota. Previous studies have demonstrated that intestinal bacterial translocation could be a normal physiological event, and under healthy conditions the enteric organisms can move across the “intact” intestinal epithelium into normally sterile tissues including blood, contributing to development of systemic and/or organ immune system (Brenchley and Douek, 2012; Wiest et al., 2014). Quite contrary to the traditional concept that circulating blood is sterile in health, the blood may harbor a commensal microbiome, which is likely to be altered during disease.

In spite of compelling evidence indicating the existence of blood microbiome in SAP patients, our study has several limitations. The primary limitation is that the results are based on sequencing of 16S rRNA genes, which is not sufficient to determine if a live microbiota is present in the circulation. It is required for metatranscriptomic studies to validate the presence/absence of a live bacterial microbiome in the blood. Another limitation is that 16S rDNA-based community profiling only provides information on the microbiota composition. Future studies will be needed to move from taxonomic description to detailed, metagenomics-based functional characterizations of the microbiota. Additionally, the number of individuals sampled in our study is relatively small, and thus it will be important to validate these observations with increasingly larger and more sophisticated human cohort studies.

In summary, we have presented compelling evidence that the blood contains a diverse bacterial microbiome in SAP patients. We also have identified the unique compositional signature of the blood and neutrophil-associated microbiomes that could distinguish SAP patients from healthy controls. More importantly, our data reveal potential links between the alterations in the blood and neutrophil-associated microbiomes and the immunological disorders in SAP patients. Yet, the possible involvement of blood microbiome variations in the development of systemic infection remains uncertain in SAP patients. Here we have only just begun to delineate the outline of the blood microbiome, and further investigations would likely provide novel insights into its roles in health and diseases.

## Author contributions

QL, CW, and JL conceived of the work. QL and CW designed experiments and analyzed data. CW, CT, XZ, and QH enrolled the patients, collected samples, performed the experiments, and prepared the figures and tables. CW wrote the article and revised it critically for important intellectual content. QL reviewed and revised the manuscript. All authors read and approved the final version of the manuscript for submission.

### Conflict of interest statement

The authors declare that the research was conducted in the absence of any commercial or financial relationships that could be construed as a potential conflict of interest.
