# Supplementary material for: Identification and Characterization of Blood and Neutrophil-Associated Microbiomes in Patients with Severe Acute Pancreatitis Using Next-Generation Sequencing
Source: Front Cell Infect Microbiol. 2018 Jan 23;8:5. doi: 10.3389/fcimb.2018.00005 (PMC5790034; doi:10.3389/fcimb.2018.00005)
Supplement: Supplementary file 1 [file Presentation1.pdf]

Supplementary Material

Identification and Characterization of Blood and Neutrophil-Associated Microbiomes in Patients with Severe Acute Pancreatitis Using Next-Generation Sequencing

Qiurong Li[*] [†], Chenyang Wang[†], Chun Tang[†], Xiaofan Zhao, Qin He and Jieshou Li

Research Institute of General Surgery, Jinling Hospital, Medical School, Nanjing University, Nanjing 210002, China

[*]**Correspondence:** Qiurong Li, liqiurongjue@126.com

[†]These authors have contributed equally to this work.

Supplementary Material includes:

Supplementary Figures 1 to 13, Supplementary Tables 1 and 2.

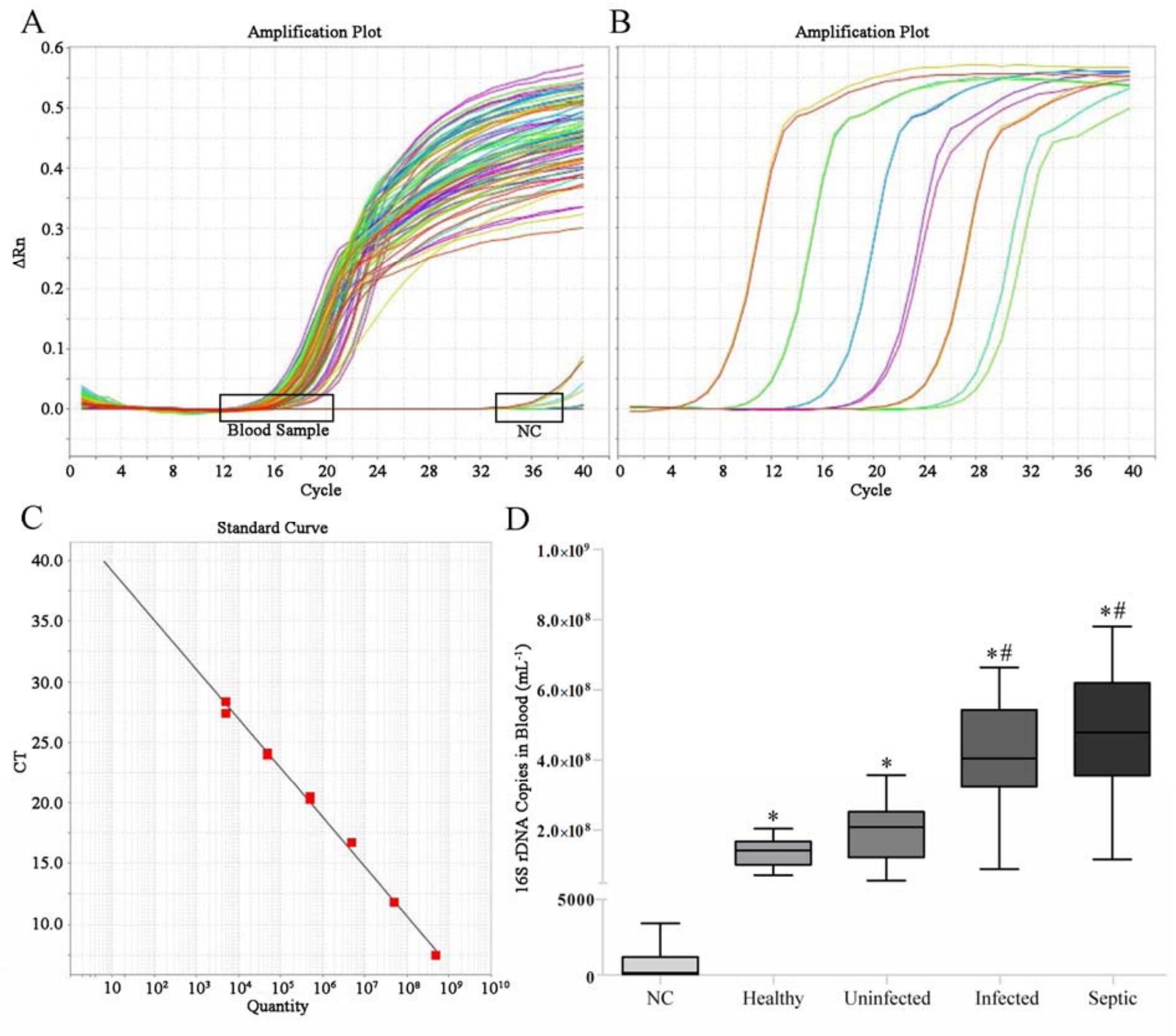

Supplementary Figure 1 Determination of the bacterial 16S rDNA in blood samples by quantitative polymerase chain reaction (qPCR) assay. (**A**) Amplification plots of qPCR obtained from blood samples and negative controls (NC). The assays were performed on the AB7500 real time PCR system (Life Technologies, CA). Amplification plots (**B**) and standard curve (**C**) generated from a series of 10-fold dilutions from 5×10^3 to 5×10^8 of 16S rRNA gene copies per reaction. (**D**) Comparison of the bacterial 16S rDNA concentrations in blood samples between patients and healthy controls (HC). The NC indicates the background signal from the reagents and consumables. * *P*<0.01, *vs*. NC; # *P*<0.01, *vs*. HC.

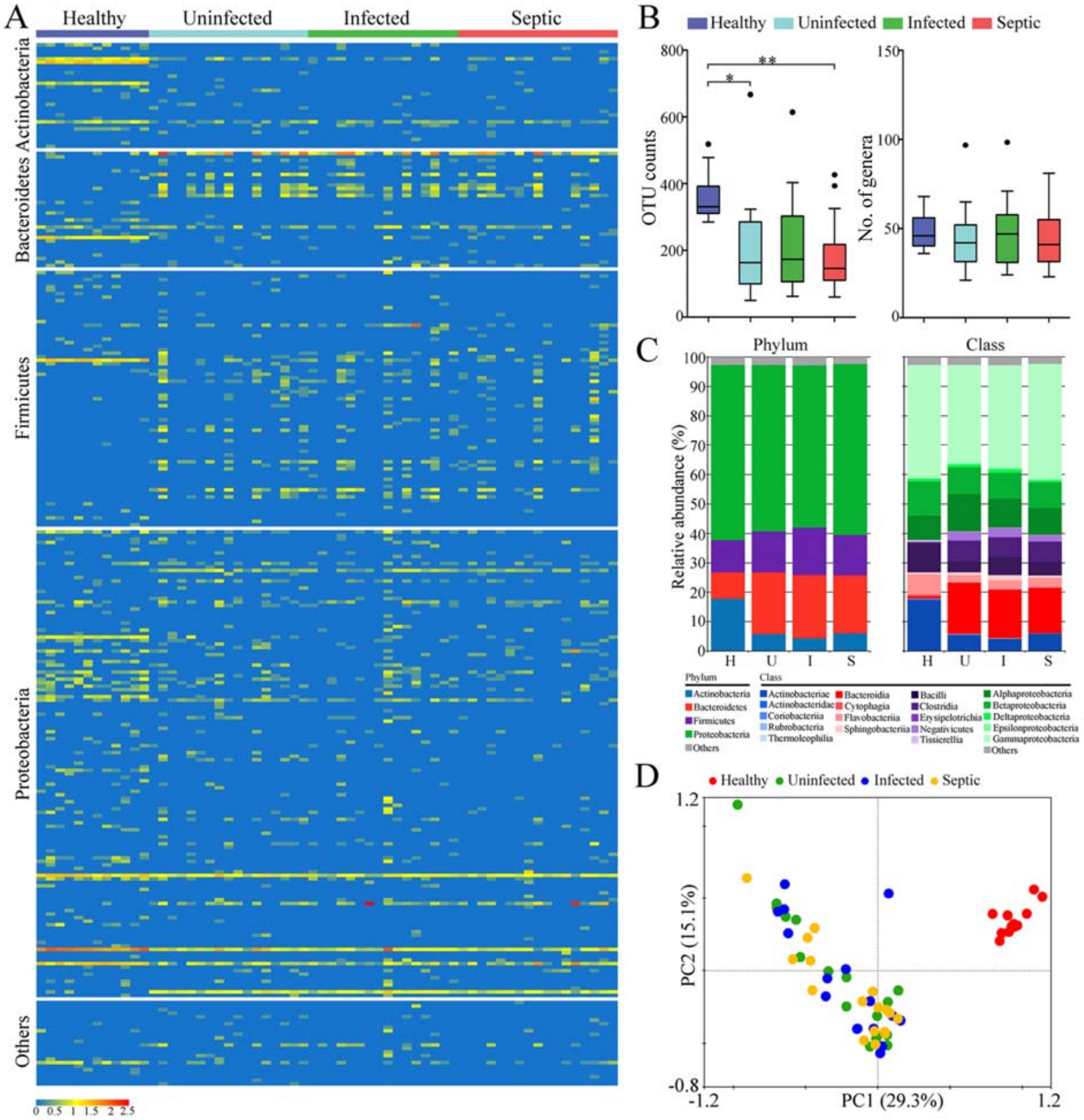

Supplementary Figure 2 Composition of the blood bacterial microbiome both in SAP patients with sepsis and in healthy subjects. (**A**) Heatmap exhibiting the bacterial genera presented in the blood of each individual by next-generation sequencing of 16S rDNA amplicons. The data represent the Log10 values of the operational taxonomic unit (OTU) counts for each genus. (**B**) Comparative analysis of the counts of observed OTUs and bacterial genera among groups. *P<0.05; **P<0.01. (**C**) Bacterial composition of the blood microbiota at the phylum and class levels. The letters “H”, “U”, “I” and “S” represent the healthy, uninfected, infected and septic groups, respectively. (**D**) Principal component analysis (PCA) of weighted UniFrac distances, based on the relative abundance of each genus, showing the difference of the microbial community structures.

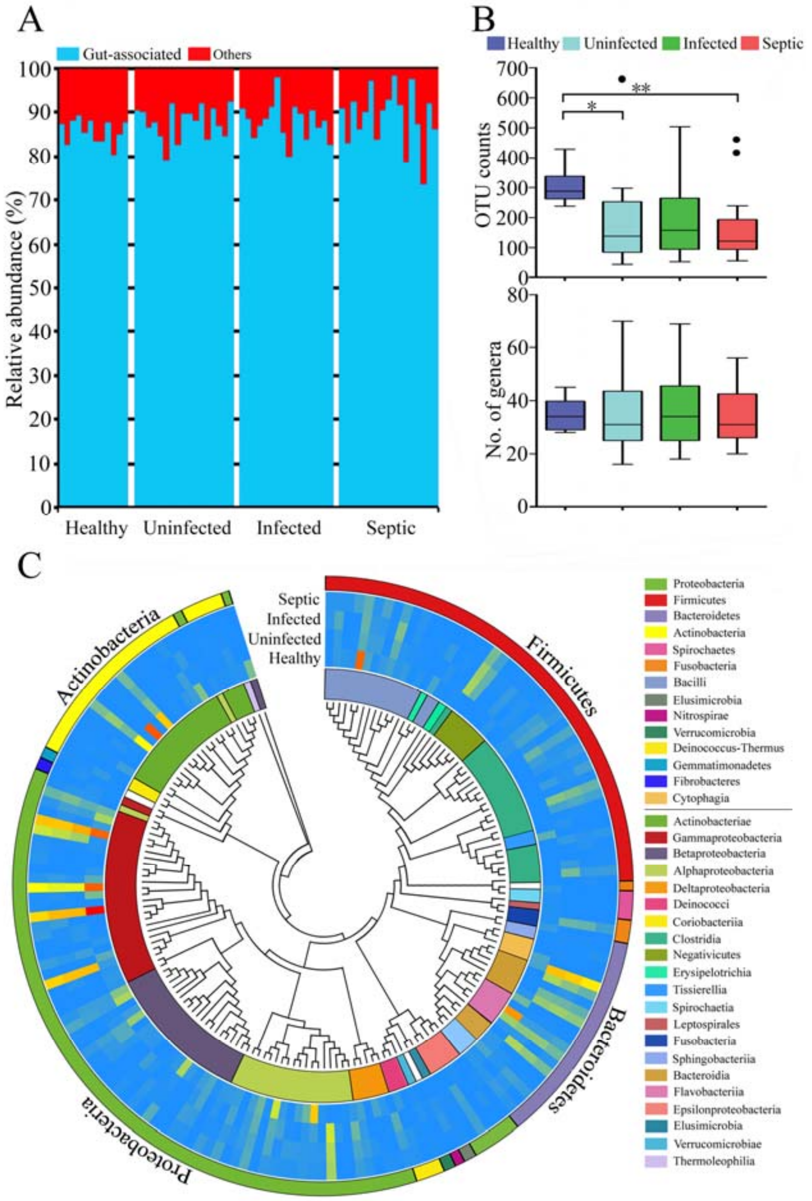

Supplementary Figure 3 The putative gut-derived organisms in blood microbiomes both in SAP patients and in healthy subjects. (**A**) Relative abundance of the blood microorganisms presumably derived from the gut or other environmental sites. (**B**) Comparison of the counts of putative gut-derived bacterial taxons between groups. *P<0.05; **P<0.01. (**C**) The phylogenetic analysis of gut-associated bacterial composition of the blood microbiota. The data presented in the heatmap represent the means of the Log10 values of the operational taxonomic unit (OTU) counts from each group.

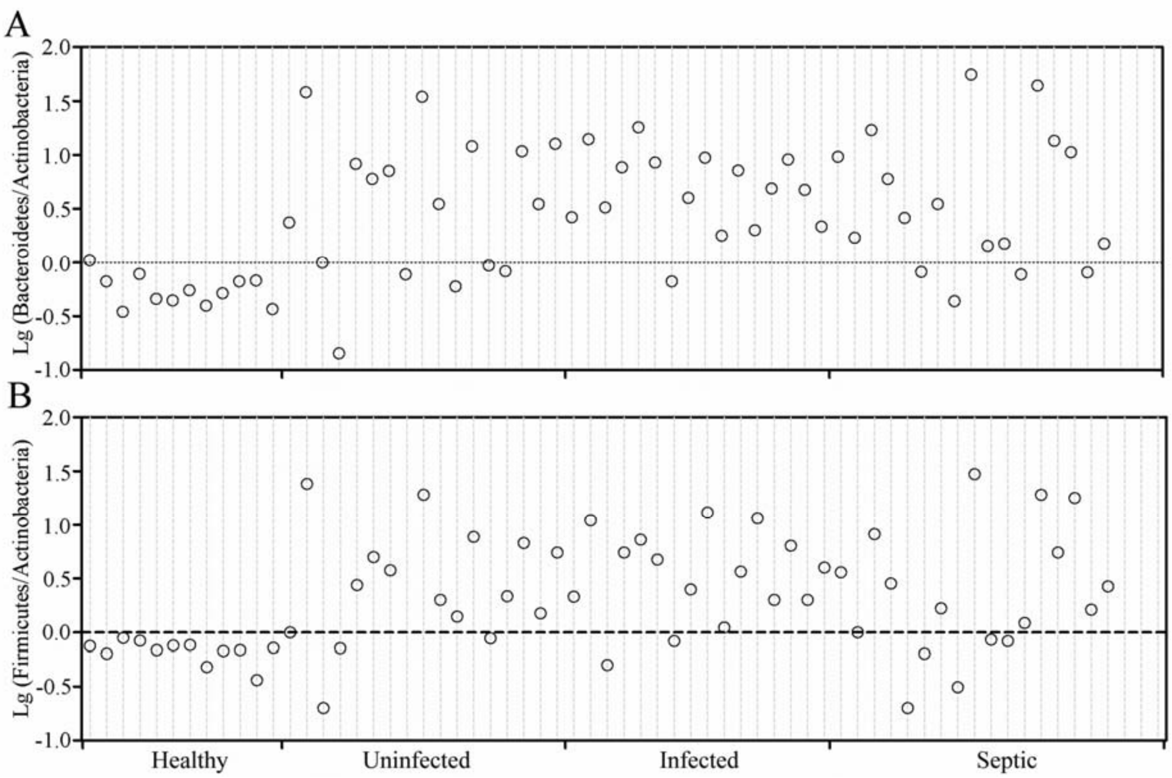

Supplementary Figure 4 Changes in the predominant bacterial phyla in blood microbiomes of SAP patients. The graphs showing shifts of the relative abundance of Bacteroidetes (**A**) and Firmicutes (**B**) compared to that of Actinobacteria. Each circle represents the Log10 value of the ratio of the relative abundance of both phyla.

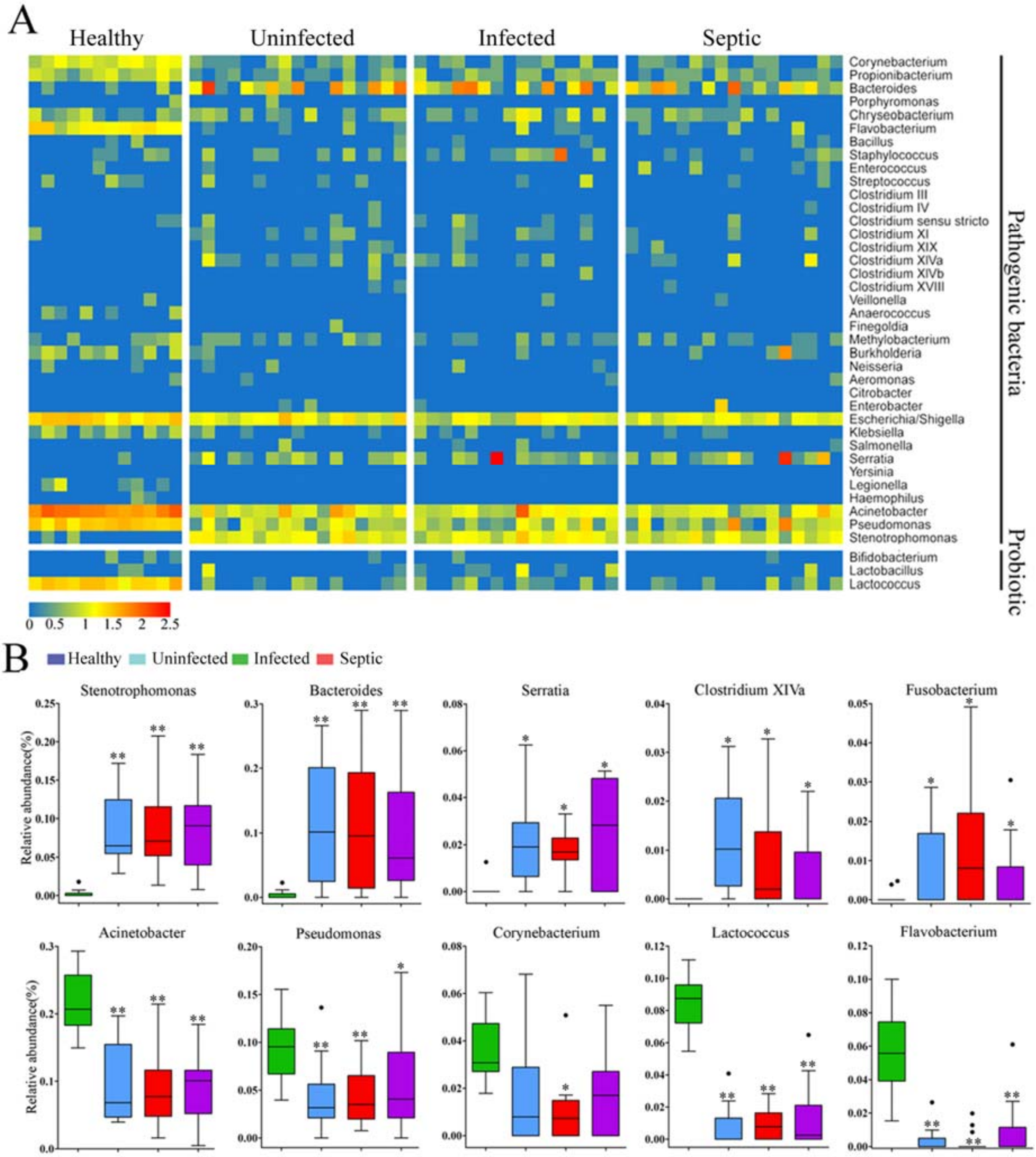

Supplementary Figure 5 Changes in specific bacterial taxa in the blood microbiomes. (**A**) Heatmap showing the composition of the potentially pathogenic and probiotic bacterial genera in the blood. (**B**) Changes of some keystone bacterial genera presumably associated with the gut. * *P* <0.05; ** *P* <0.01, *vs*. Healthy.

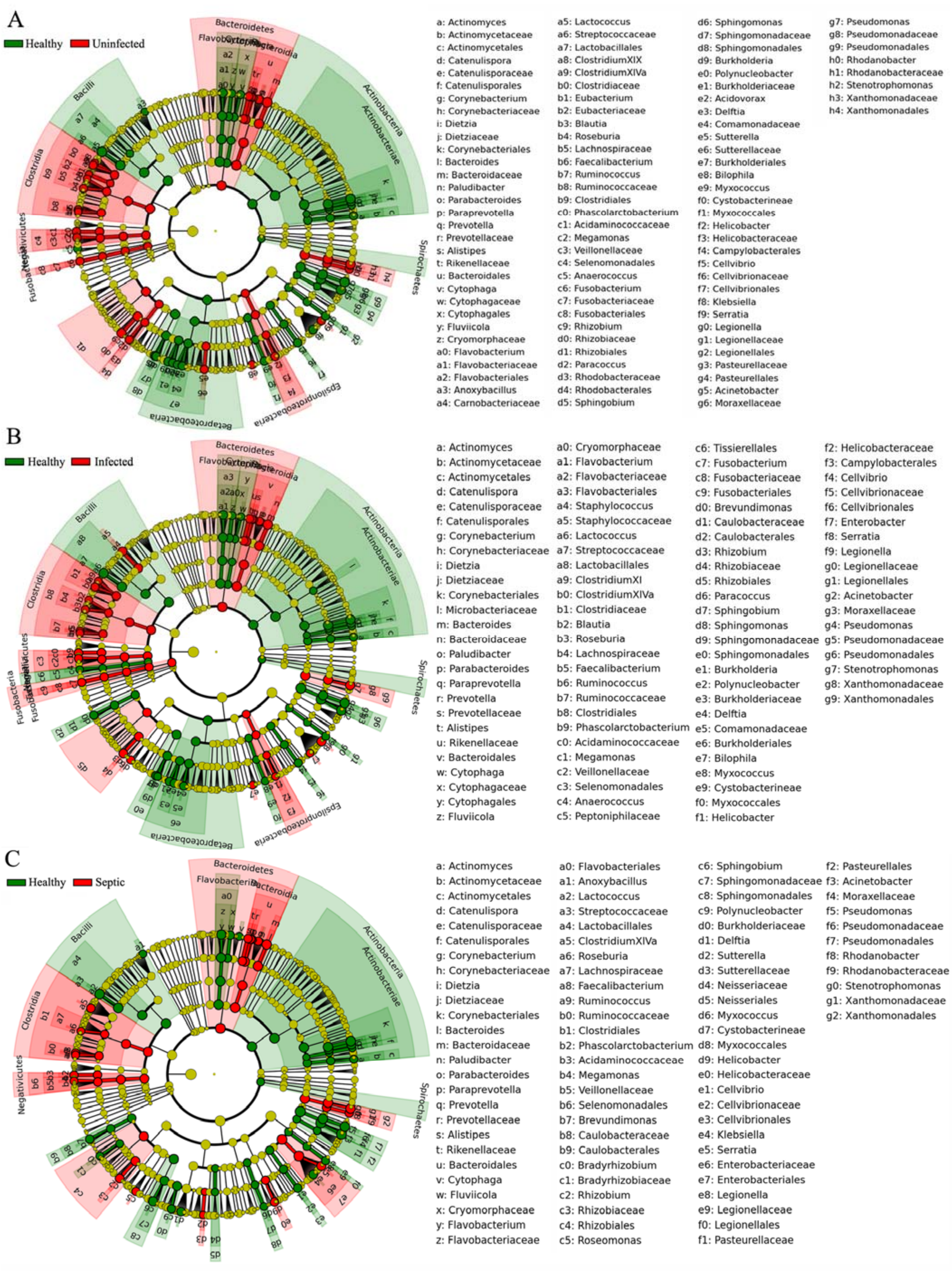

Supplementary Figure 6 LEfSe analyses based on OTUs characterize the differences in the blood microbiome composition between the patients and healthy subjects.

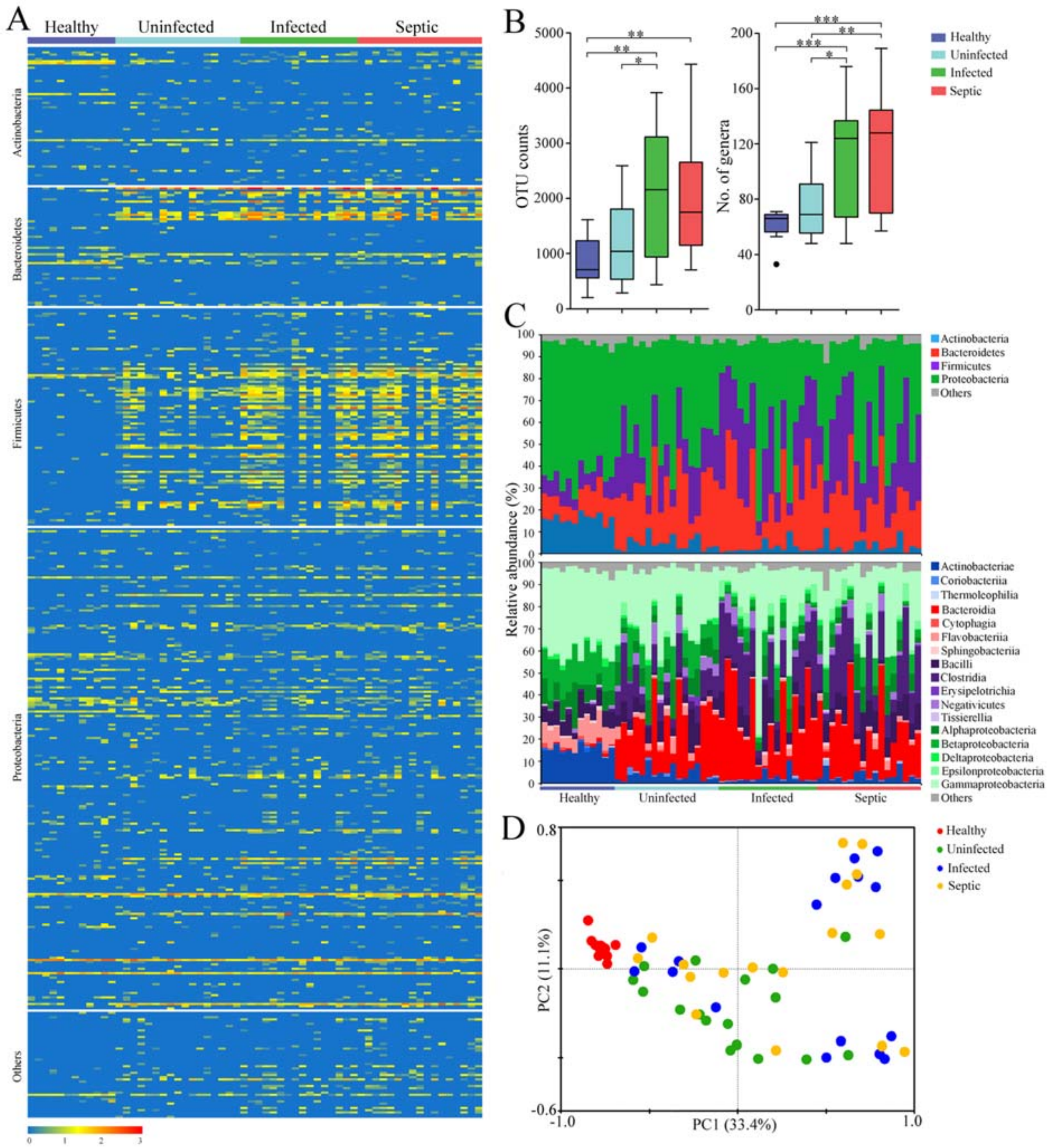

Supplementary Figure 7 Comparison of the neutrophil-associated microbiomes (NAMs) between patients and healthy controls. (**A**) Heatmap showing the abundance of the bacterial genera identified by taxonomic classification. The data represent the Log10 values of the operational taxonomic unit (OTU) counts for each genus. (**B**) Differences in the OTU and genera counts of the NAMs between groups. * *P* <0.05; ** *P* <0.01; *** *P* <0.001. (**C**) Measurement of the predominant bacterial composition in the blood microbiotas at the phylum and class levels. (**D**) Principal component analysis (PCA) of weighted UniFrac distances, based on the relative abundance of each genus, indicating the difference of the microbial community structures in the NAMs among groups.

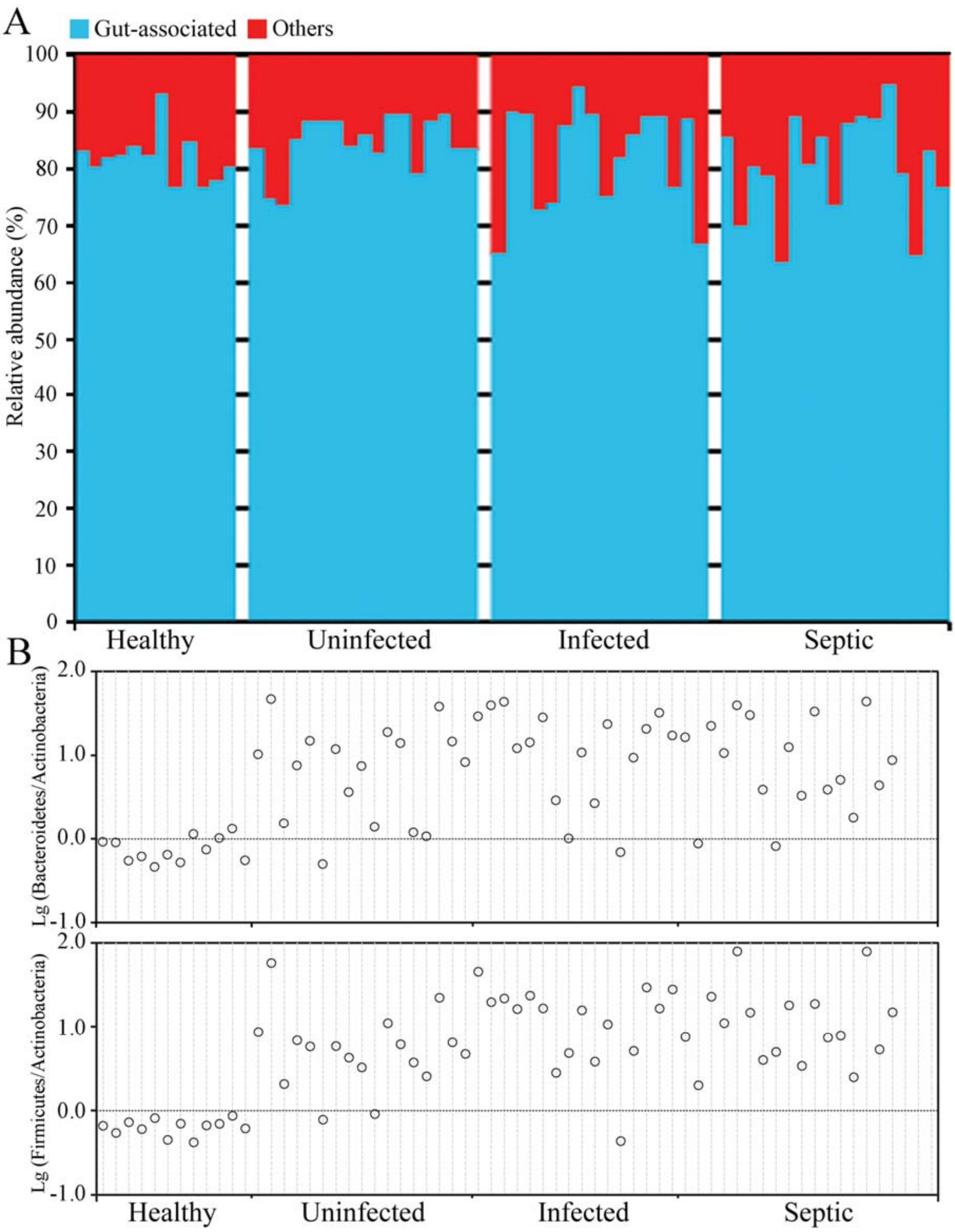

Supplementary Figure 8 Putative gut-derived organisms in the neutrophil-associated microbiomes of the neutrophils both in SAP patients and in healthy subjects. (**A**) Relative abundance of the neutrophil-associated microorganisms presumably derived from the gut and other environmental sites. (**B**) The graphs showing shifts of the relative abundance of Bacteroidetes and Firmicutes compared to that of Actinobacteria. Each circle represents the Log10 value of the ratio of the relative abundance of both phyla.

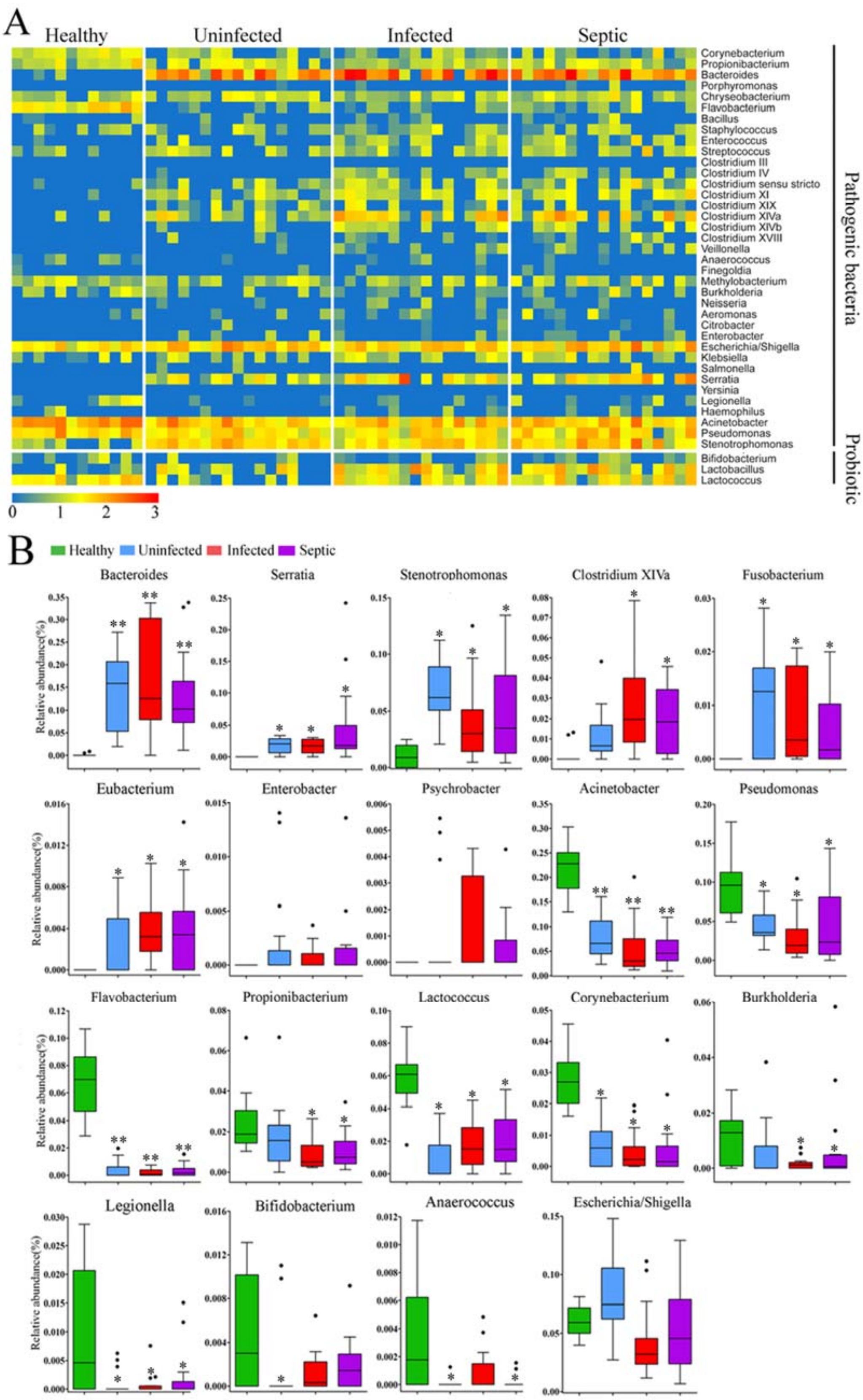

Supplementary Figure 9 Alterations in specific bacterial taxa in the neutrophil-associated microbiomes. (**A**) Heatmap showing the composition of the potentially pathogenic and probiotic bacterial genera in the neutrophils. (**B**) Changes of some keystone bacterial genera presumably associated with the gut. * *P* <0.05; ** *P* <0.01, *vs*. Healthy.

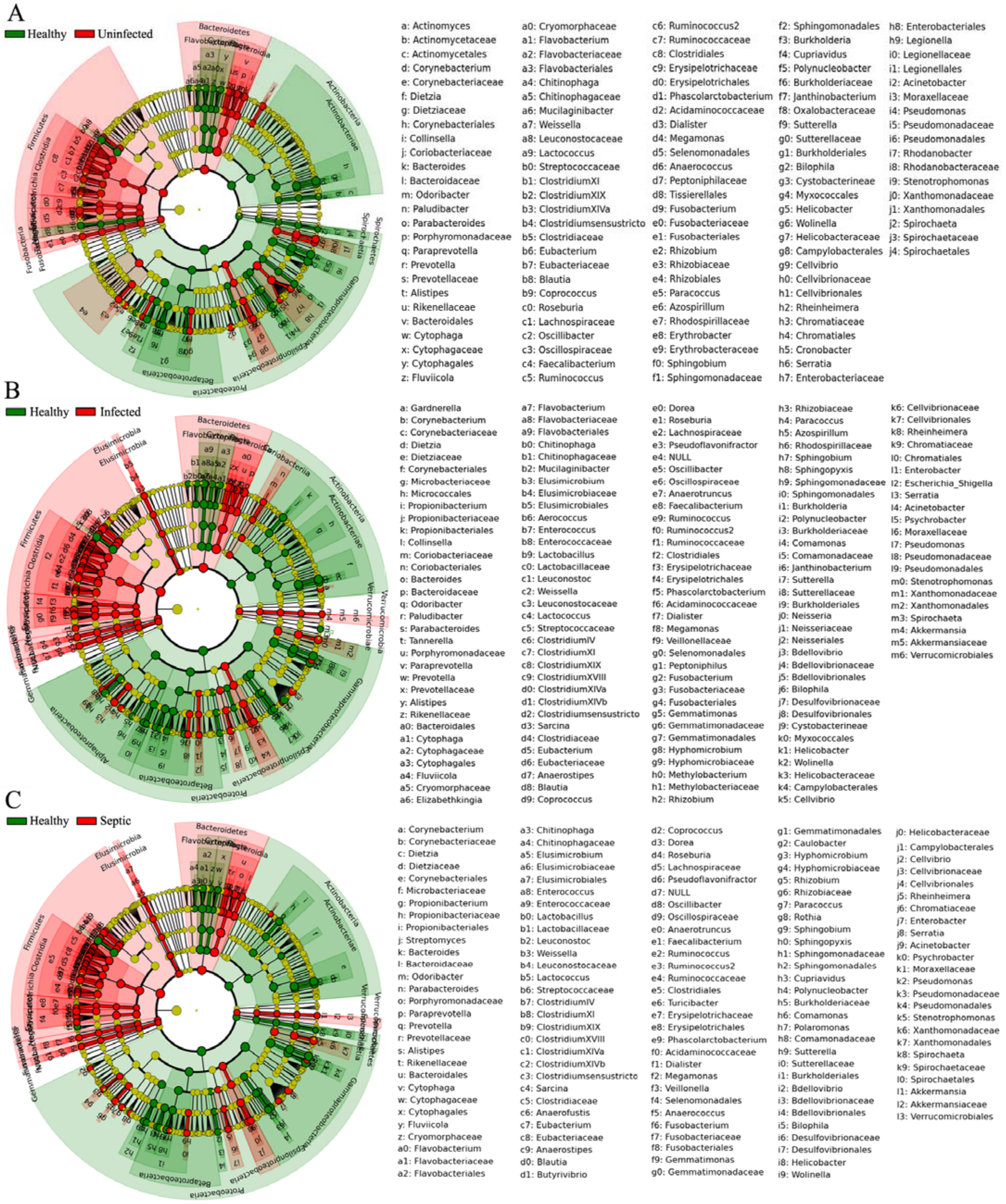

Supplementary Figure 10 LEfSe analyses of the gut-associated organisms in neutrophil-associated microbiomes from the patients and healthy subjects.

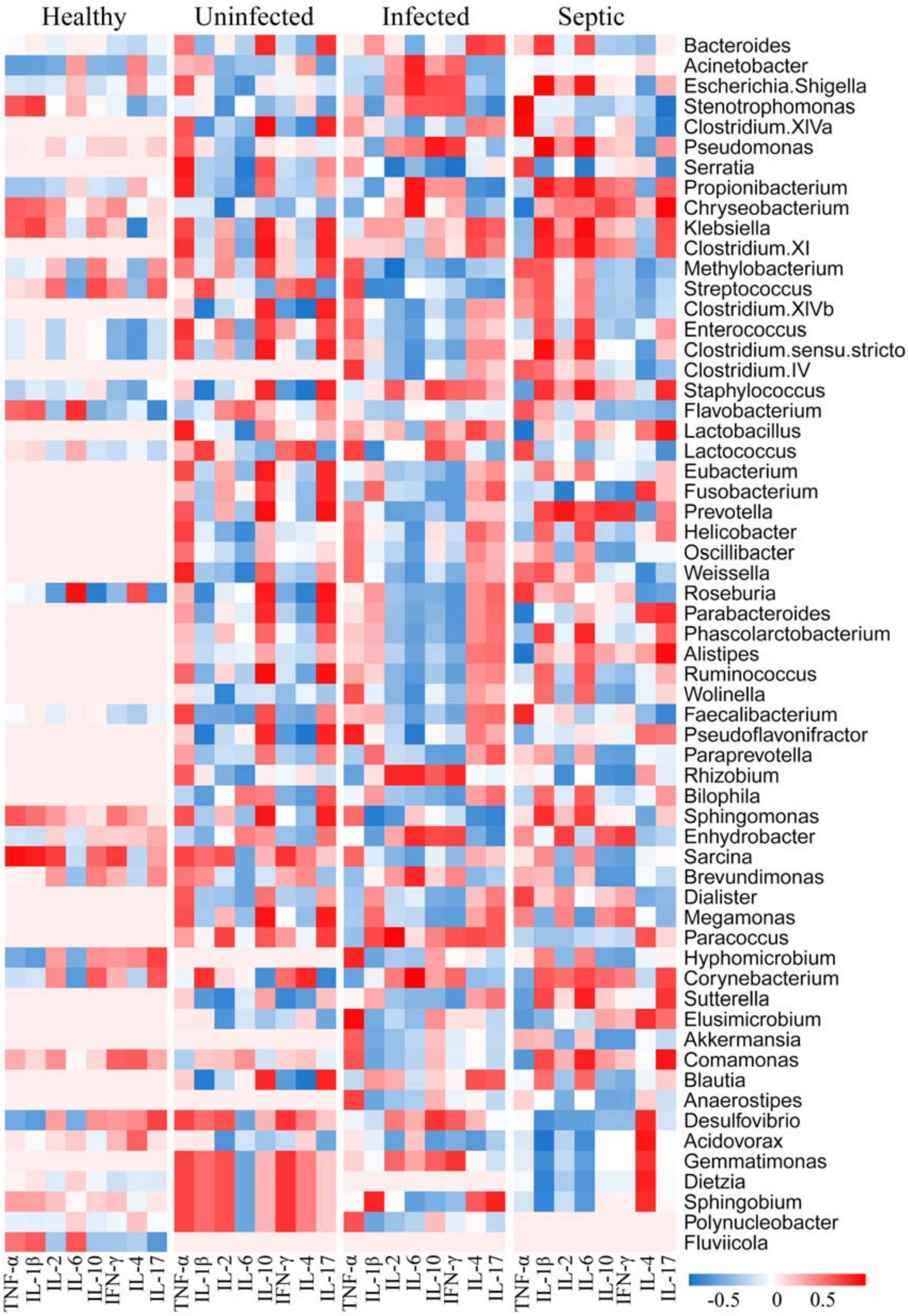

Supplementary Figure 11 Correlations between the predominant bacterial genera in the neutrophils and the levels of serum cytokines. The data presented in the heatmap indicate the correlation coefficient between variances.

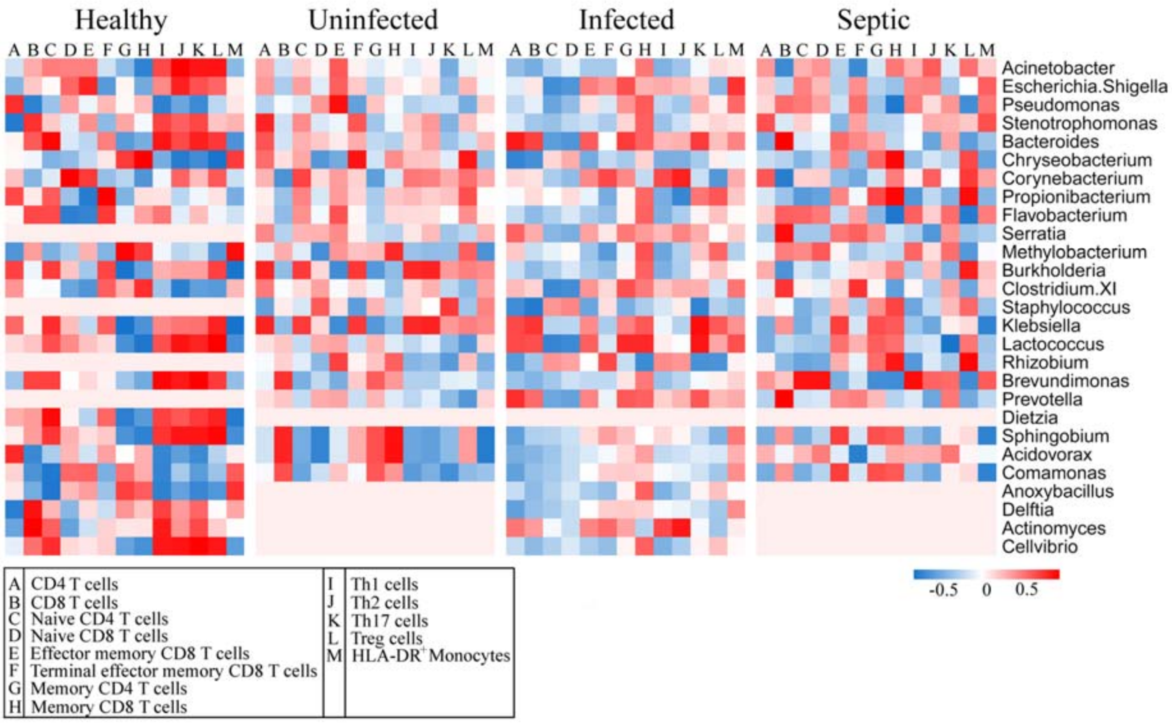

Supplementary Figure 12 Correlations of the predominant bacterial genera in the blood with the subpopulations of peripheral lymphocytes. The data presented in the heatmap indicate the correlation coefficient between variances.

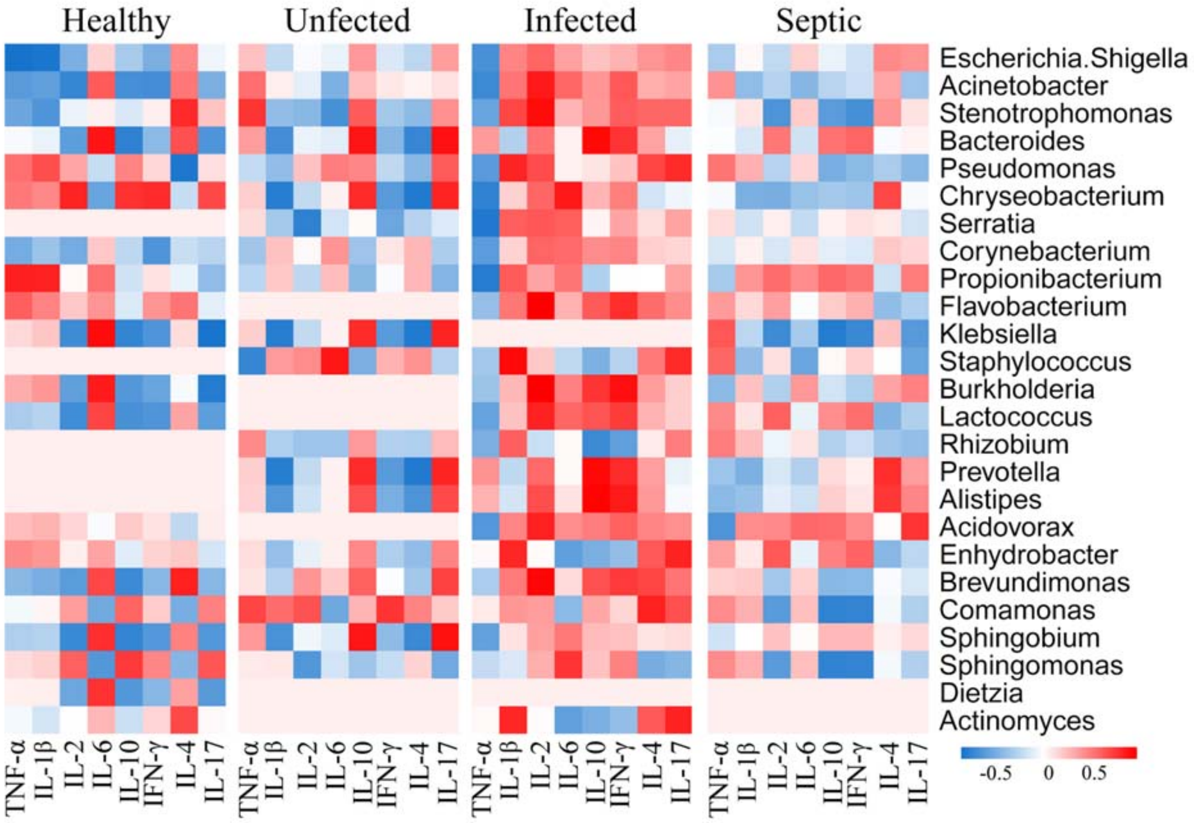

Supplementary Figure 13 Correlations between the blood predominant bacterial genera and the levels of serum cytokines. The data presented in the heatmap indicate the correlation coefficient between variances.
